# A comprehensive overview of bull sperm-borne small non-coding RNAs and their diversity across breeds

**DOI:** 10.1186/s13072-020-00340-0

**Published:** 2020-03-30

**Authors:** Eli Sellem, Sylvain Marthey, Andrea Rau, Luc Jouneau, Aurelie Bonnet, Jean-Philippe Perrier, Sébastien Fritz, Chrystelle Le Danvic, Mekki Boussaha, Hélène Kiefer, Hélène Jammes, Laurent Schibler

**Affiliations:** 1 R&D Department, ALLICE, 149 rue de Bercy, 75012 Paris, France; 2 Université Paris-Saclay, AgroParisTech, INRAE, GABI, 78350 Jouy-en-Josas, France; 3 Université Paris Saclay, UVSQ, INRAE, BREED, 78350 Jouy en Josas, France; 4 Ecole Nationale Vétérinaire d’Alfort, BREED, 94700 Maisons-Alfort, France

**Keywords:** sncRNA, miRNA, isomiR, piRNA, tRNA, Sperm, Bull

## Abstract

**Background:**

Mature sperm carry thousands of RNAs, including mRNAs, lncRNAs, tRNAs, rRNAs and sncRNAs, though their functional significance is still a matter of debate. Growing evidence suggests that sperm RNAs, especially sncRNAs, are selectively retained during spermiogenesis or specifically transferred during epididymis maturation, and are thus delivered to the oocyte at fertilization, providing resources for embryo development. However , a deep characterization of the sncRNA content of bull sperm and its expression profile across breeds is currently lacking. To fill this gap, we optimized a guanidinium–Trizol total RNA extraction protocol to prepare high-quality RNA from frozen bull sperm collected from 40 representative bulls from six breeds. Deep sequencing was performed (40 M single 50-bp reads per sample) to establish a comprehensive repertoire of cattle sperm sncRNA.

**Results:**

Our study showed that it comprises mostly piRNAs (26%), rRNA fragments (25%), miRNAs (20%) and tRNA fragments (tsRNA, 14%). We identified 5p-halves as the predominant tsRNA subgroup in bull sperm, originating mostly from Gly and Glu isoacceptors. Our study also increased by ~ 50% the sperm repertoire of known miRNAs and identified 2022 predicted miRNAs. About 20% of sperm miRNAs were located within genomic clusters, expanding the list of known polycistronic pri-miRNA clusters and defining several networks of co-expressed miRNAs. Strikingly, our study highlighted the great diversity of isomiRs, resulting mainly from deletions and non-templated additions (A and U) at the 3p end. Substitutions within miRNA sequence accounted for 40% of isomiRs, with G>A, U>C and C>U substitutions being the most frequent variations. In addition, many sncRNAs were found to be differentially expressed across breeds.

**Conclusions:**

Our study provides a comprehensive overview of cattle sperm sncRNA, and these findings will pave the way for future work on the role of sncRNAs in embryo development and their relevance as biomarkers of semen fertility.

## Background

Sperm is classically seen as a transcriptionally inactive vehicle [1] that delivers the paternal haploid genome to the oocyte. Yet, an increasing number of studies in several species have shown that mature ejaculate sperm carry thousands of RNAs, including messenger RNAs (mRNAs), long non-coding RNAs, transfer RNAs (tRNAs), ribosomal RNAs (rRNAs) and small non-coding RNAs (sncRNAs), especially microRNAs (miRNA), antisense RNAs, and Piwi-interacting RNAs (piRNA). Since transcription is mostly silenced in sperm, these RNAs are often considered to be remnant transcripts, produced during previous spermatogenesis stages [2–4]. However, a comparison of sperm and testes in horses instead suggested that the repertoire of sperm RNAs is not a random spermatogenesis remnant, but rather a selectively retained and functionally coherent collection of RNAs [5]. In addition, some studies in mouse have provided evidence for sncRNA regulation as sperm mature along the epididymis [6, 7], with sncRNA being transferred to sperm during post-testicular maturation, possibly through epididymosomes [8]. The functional significance of sperm sncRNAs, if any, is still a matter of debate, and future studies are required to shed light on their potential biological role. However, there is now some evidence of a role in fertilization and embryo development. Indeed, mouse sperm-borne RNAs are delivered to the oocyte at fertilization [9] and may provide essential resources for the first steps of embryo development [10]. In agreement with this hypothesis, sperm-borne miRNA-34c has been shown to be delivered into the mouse zygote during fertilization and play a role in the first cell division [11]. More generally, paternal miRNAs and/or endo-siRNAs have been identified as crucial for fertilization and preimplantation embryonic development [12] and post-natal outcome [13]. In addition, involvement of sperm sncRNA has been demonstrated in epigenetic inheritance, including miRNAs [14, 15] as well as piRNAs and tRNAs [16, 17].

Unfortunately, the study of sperm sncRNAs is challenging due to technical issues related to sperm RNA isolation, including low RNA content and resistance to lysis by detergents [9, 18]. Additionally, differences in sperm morphology and chromatin condensation may impair the interspecies use of sperm RNA isolation protocols. Consequently, highly variable sncRNA content has been obtained using different RNA extraction procedures, and until very recently only a small number of comprehensive studies were conducted. For instance, SpermBase was established in 2016 to publish RNA-seq data on mRNAs and sncRNAs in mouse, rat, rabbit, and human total sperm as well as sperm heads [19]. Among sncRNAs, 18S and 28S rRNA fragments were reported to be abundant in sperm [20], as were tRNA-derived fragments (tsRNAs) [21]. Sperm tsRNAs are mainly fragments of the 5′ end of tRNA, ranging in length from 20 to 35 nt, and are produced by specific cleavage of tRNA at either D-Loop, Anticodon-Loop or T-Loop by RNase Z, Dicer or Angiogenin [21, 22]. They were recently proposed to mediate, at least in part, transgenerational effects associated with parental diet [16, 23]. In addition, sperm tsRNAs are subjected to numerous RNA modifications and edits that contribute to their stability (for review see [24]). Detailed studies of the sperm miRnome have also been undertaken in several species, including humans [25], mice [26, 27], horses [28], pigs [29] and cattle [30, 31]. Extensive variations in length and sequence composition of miRNAs were detected by deep sequencing. These so-called isomiRs are thought to be produced via imprecise and alternative cleavage by Drosha or Dicer along the miRNA biogenesis pathway [32] and do not represent transcription or sequencing errors [33]. Shortening of miRNAs may also arise by exonuclease trimming of the end [34]. Conversely, the post-transcriptional addition of one or more nucleotides by template-independent nucleotidyl transferases may increase miRNA length [35]. In particular, seven nucleotidyl transferases have been implicated in human isomiR biogenesis, especially uridyltransferases and adenyltransferases [36], including PAPD4 which is considered to be the primary miRNA adenylating enzyme [37]. Polymorphisms within the internal canonical sequences have also been described in isomiRs expressed at low levels [38], resulting either from genetic polymorphism or from A–I editing, i.e., the hydrolytic deamination of adenosine to inosine in double-stranded RNA [36]. A growing body of evidence indicates that these changes may affect isomiR stability or influence target selection [36, 37]. IsomiRs have been shown to be non-randomly distributed and expressed in a developmental- and tissue-specific manner in several human tissues [39]. Their broader biological significance is yet to be fully resolved, but some studies suggest that alteration in isomiR profiles, rather than in the overall miRNA abundance, is of biological significance, correlating for instance with cancer progression [40]. In cattle, several miRNAs have been shown to be differentially expressed between high- and low-fertility bulls [41, 42], as well as between high- and low-motility sperm [30].

This study was thus designed to unravel the sncRNA content from frozen bull sperm, focusing on tsRNAs, miRNAs, and their structural variations (called “isomiRs” by Morin et al. [43]), as well as the diversity of their expression profiles according to breeds. To do so, we optimized a guanidinium–Trizol total RNA extraction protocol for frozen bull sperm to ensure good sample quality and reproducibility.

## Results

### RNA extraction and NGS sequencing

Selected samples had normal sperm quality profiles, consistent with routine results obtained in semen production centers (data not shown). Contamination with somatic cells was negligible, as confirmed by microscopy (less than 1 somatic cell per 1000 sperm cells) and side-scatter profiles obtained by flow cytometry (data not shown). In addition, no RNAs could be extracted from the extender used by semen production centers to dilute the sperm cells. Altogether, these results indicate that the identification of sperm sncRNA content is not jeopardized by contaminants.

On average, 57.6 ± 12.9 ng of total RNA could be prepared from 31.2 ± 7 million thawed sperm cells. Technical variation was evaluated based on multiple analyses of a standard ejaculate, showing good reliability with a coefficient of variation less than 10% in the amount of total RNA prepared using the protocol. Typical electrophoretic profiles were obtained, with no evidence of 18S and 28S rRNAs. RT-qPCR was performed to validate RNA quality and concentrations on a subset of 3 samples for each extraction batch, leading to consistent amplification results (Ct in the range of 20–21 starting from 5 ng of total RNA) and single peak melt curves, indicating that a single, specific product had been produced (Additional file 1: Fig. S1).

Sequencing resulted in 1,444,722,663 total raw sequence reads. About 36.1 million reads were obtained on average (± 4.5 million, min = 28.3 and max = 49.5) for the 40 libraries. The overall data quality was good, with > 97% of the data having a Q-score over 30 (i.e., a base-calling accuracy of 99.9%). Typical examples of FastQC quality control plots are provided in Additional file 2: Fig S2a. After trimming sequencing adapters, read length distribution revealed two main peaks representing mostly microRNAs or siRNAs (~ 18–26 nt) and intermediate sequences (28–32 nt), including piRNAs and tsRNAs (Additional file 2: Fig S2b). About 72% of sequences could be mapped unambiguously to the cattle reference genome on average, while 15% were outmapped (i.e., mapped to abundant sequences such as polyA and polyC homopolymers or other repetitive sequences) and 13% were unmapped (Table 1).

**Table 1:** Mapping statistics across the 40 libraries

	Mapped (72%)	Outmapped (15%)	Unmapped (13%)	Reads
Reads no.	1,038,383,839	223,342,595	181,996,229	1,443,722,663
Mean	25,959,596	5,583,565	4,549,906	36,093,067
Std. deviation	3,542,949	1,932,607	4,370,783	4,483,073

### The overall small RNA content of bull sperm

**Fig. 1:**
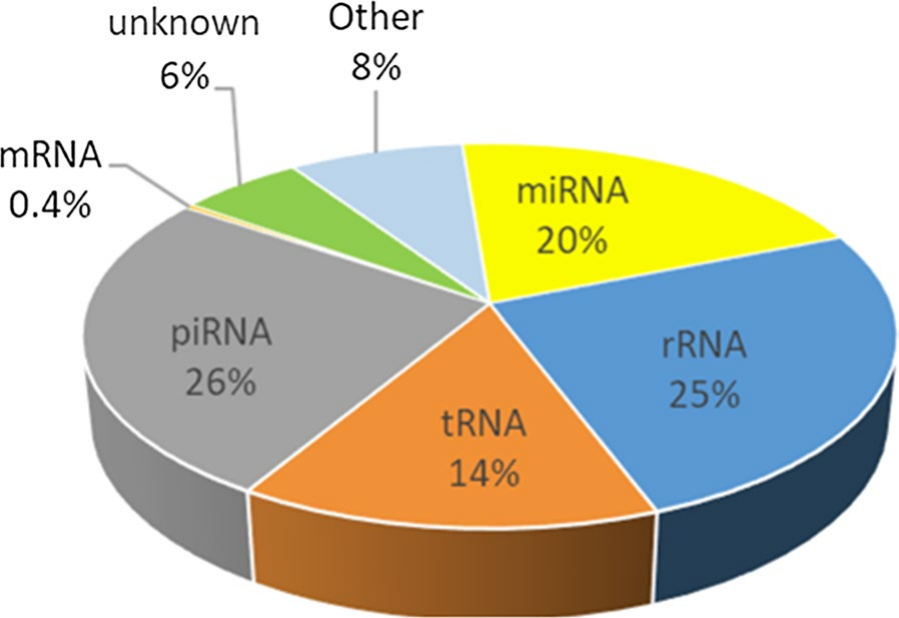
sRNA content of bull sperm. miRDeep2 was used to identify miRNAs, and the remaining reads were annotated (e.g., as rRNA, tRNA, piRNA) using several sncRNA databases. The percentages of total number of reads for each category are represented as a pie chart

Reads were analyzed and categorized as described in the Material and Methods. First, 701 known and 2022 putative miRNAs were identified using miRDeep2 (Additional file 3: Table S1). The remaining reads were then annotated using several mRNA and sncRNA databases, highlighting the diversity of sncRNA in cattle sperm (Additional file 3: Tables S2–S6). As summarized in Fig. 1, sperm RNA contains mainly piRNAs (26%), rRNAs (25%), miRNAs (20%), tRNAs (14%), other sncRNAs (8%, including mainly Signal Recognition Particle (SRP) RNA, fragments of lncRNAs, Y_RNA and snRNA) and 6% of unknown sequences. In addition, about 27,655 reads (with a majority being 50 nt in length) were identified as mRNA fragments originating from 3510 genes, of which 95% were already described in human or mouse sperm (http://spermbase.org). The gene coverage was usually low (7.7 ± 3.1 different unique reads per gene on average, preferentially clustered at the 5p end of the gene), but a few genes were found to be covered by well-distributed unique reads (37 genes with more than 100 different reads) and have high read counts (see for instance the *AKAP1* and *PRM1* IGV profiles in Additional file 4: Fig S3). A gene ontology analysis of these genes revealed several relevant enriched biological terms, including male gamete generation (GO:0048232), spermatogenesis (GO:0007283), and gamete generation (GO:0007276).

### Bull sperm contains abundant rRNA-derived small RNAs

**Fig. 2:**
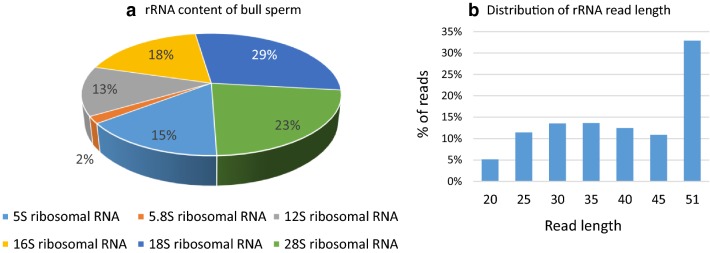
Bull sperm rRNA content. The proportion of reads annotated for different rRNAs is shown (**a**) as well as the distribution of read length (**b**)

About 25% of reads were annotated as rRNAs, mainly the 16S (18%), 18S (29%) and 28S (23%) rRNAs, as illustrated in Fig. 2a. Though highly abundant, these rRNAs appeared to be fragmented, with two-thirds being less than 45 nt in length (Fig. 2b). In addition, the distribution of reads along rRNA transcripts revealed a series of peaks and read-poor sub-regions, especially for 18S and 28S rRNAs (Additional file 5: Fig S4).

### Bull sperm contains abundant piRNAs

**Fig. 3:**
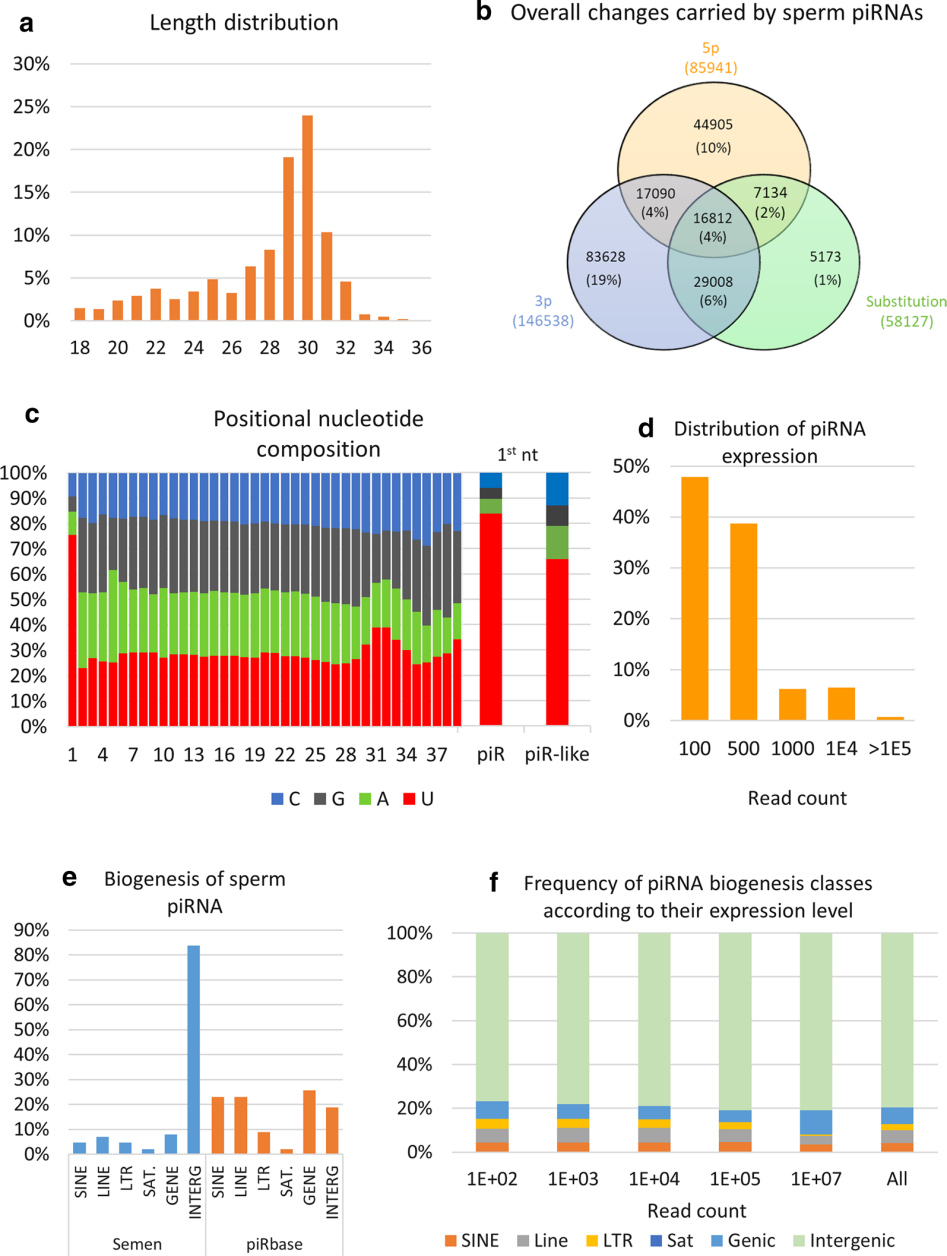
Bull sperm piRNA content. **a** Reads annotated as piRNAs showed a typical size distribution, with a majority of reads being 27–32 nt in length. **b** Among these reads, 54% matched perfectly to published piRNA sequences. The remaining reads were considered to be piRNA-like sequences, bearing mostly 3p and 5p changes (19% and 10% of reads, respectively) or a combination (17%) of 5p, 3p and/or substitutions. **c** Both piRNAs and piRNA-like sequences showed a specific nucleotide composition, with more than 84% and 66%, respectively, showing a preference for uracil at their 5p terminus. **d** The majority of piRNA are expressed at low levels (87% below 500 mapped reads), with only a few (1%) expressed above 100,000 mapped reads. **e** All piRNA biogenesis classes were identified in sperm, but sperm piRNAs were found to primarily derive from intergenic regions. **f** Gene-derived piRNAs showed a specific expression profile, with higher expression levels than the other classes

About 26% of reads were annotated by piRNAs, with a size distribution peaking at 30 nt and two-thirds of reads being in the range of 27–32 nt (Fig. 3a). Among these reads, 54% matched perfectly with a published piRNA sequence. The remaining reads were considered to be piRNA-like sequences, bearing mostly 3p and 5p changes (19% and 10% of reads, respectively) or a combination (17%) of 5p, 3p and/or substitutions (Fig. 3b). Modifications of a single nucleotide at either the 5p or 3p end were the most frequent changes (81% and 67% of changes, respectively), with deletions accounting for about 85% of changes at both the 5p and 3p ends (see Additional file 3: Table S4). As expected, piRNA reads showed a typical nucleotide composition (Fig. 3c), with strong enrichment for uridine at the first position. This “1U bias” was also observed for piRNA-like sequences, although to a lesser extent (66% compared to 84% for piRNAs). No adenine enrichment at piRNA nt 10 could be observed for both piRNA and piRNA-like sequences [44]. As shown by the distribution of expression (Fig. 3d and Additional file 3: Table S4), about 50% of piRNAs were expressed at low levels (less than 100 mapped reads), and only 7% were very highly expressed (greater than 10,000 mapped reads). According to the piRBase biogenesis classification, sperm piRNAs were found to primarily derive from intergenic (84%), genic (8%) and LINE-rich (7%) regions (Fig. 3e). Interestingly, a highly significant fourfold enrichment was observed for sperm piRNAs derived from intergenic regions compared to the whole piRBase database (see Additional file 3: Table S4). Conversely, all other classes were found to be highly under-represented in sperm. When computing the frequency of each biogenesis class according to the piRNA expression level, no particular enrichment could be observed with the exception of gene-derived piRNAs, which account for a larger proportion of highly expressed reads (Fig. 3f).

### Bull sperm contains abundant tRNA-derived small RNAs (tsRNAs)

**Fig. 4:**
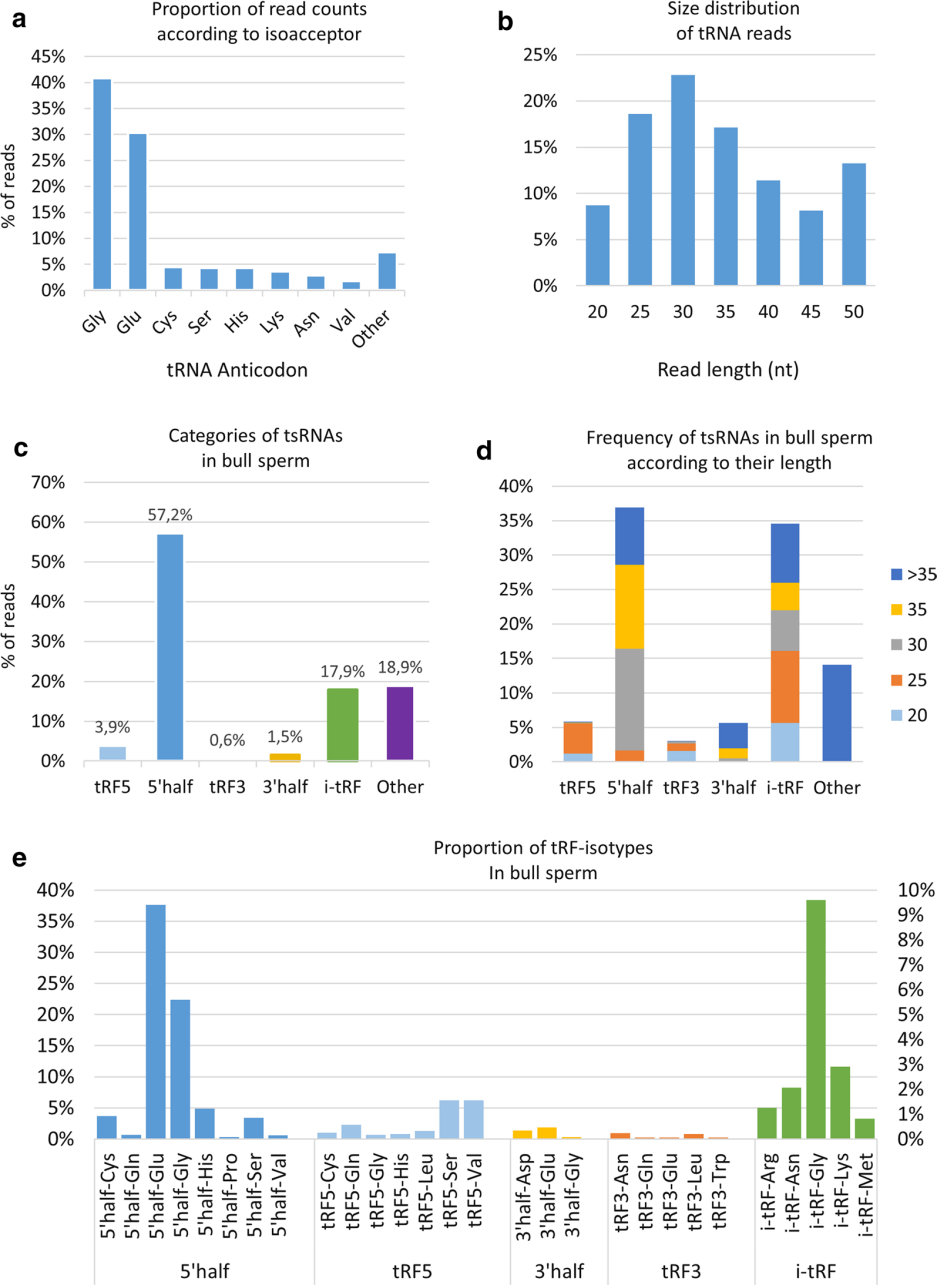
Bull sperm tsRNA content. **a** Strong expression of the Glycine and Glutamine isoacceptors was observed, as well as a high proportion of reads in the 25–35 nt range **b**. The proportion of each category and the proportion of read length in each category are shown in (**c**, **d**), respectively. To evaluate the proportion of tsRNAs isotypes, the percentage of read counts was computed for each tsRNA category and each associated anticodon (**e**). Only the most expressed tsRNAs from each category are reported on the histogram. The left axis (0–40%) refers only to 5p tRHs, which are the most expressed tsRNAs. The right axis (0–10%) refers to the other tsRNAs

A total of 61,991 sequences were annotated as tRNAs, covering the full range of isoforms associated with the 22 amino acids. However, two isoacceptors contributed to 71% of all identified tsRNA: glycine (41% of read counts) and glutamine (30% of reads counts). As illustrated in Fig. 4a, six tRNA isoforms each accounted for 2–5%, with the others together accounting for about 7% of read counts. The size of these reads was in the 18–50 nt range, with a bimodal distribution peaking at 30 nt and 50 nt (Fig. 4b).

Previous studies have shown that 20–35 nt fragments do not represent random degradation products, but instead are generated by specific mechanisms producing four main categories of tsRNAs, as illustrated in Additional file 6: Fig S5. As shown in Fig. 4c, 5p-tRHs were the most expressed tRFs, accounting for 57% of read counts, while i-tRFs and tRF5 accounted for 18% and 4%, respectively. Interestingly, nearly 65% of all i-tRFs (84% of expression) were found to be produced by mitochondria (MT) tRNAs. About 19% of reads were classified as “other” and were shown to be more than 35 nt in length (Fig. 4d). These reads were found to be mainly clustered at the 5p side of tRNAs, 75% of them starting at the 1st tRNA nucleotide. In contrast, the i-tRF group was found to be diverse in terms of size, while tRF5 and tRF3 were mainly in the 20–25 nt range. When focusing on tsRNAs in the range 20–40 nt, the most abundant tRFs were 5p-tRHs, especially Glu, Gly, His and Cys isoacceptors (Fig. 4e and Additional file 7: Fig S6).

Among i-tRFs, the most abundant were i-tRF-Gly, i-tRF-Lys and i-tRF-Asn, with distinct read length distributions: e.g., reads 20–25 nt and 25–30 nt in length contributed equally (~ 42%) to i-tRF-Gly, while 25–30 nt reads accounted for 60% of i-tRF-Lys (Additional file 7: Fig S6). The most abundant tRF5s were tRF-Ser and tRF5-Val, the majority of reads being 25 nt in length. Most tRF3s are short fragments (20 nt), with tRF3-Asn and tRF3-Leu being the most abundant. Interestingly, cleavage sites within the anticodon-loop were found to be biased towards the 5p-side of the anticodon for both tRHs (Additional file 8: Fig S7). The 4th nucleotide appeared to be a preferential cleavage site for 3p-tRHs, while 5p-tRHs are produced by cleavage between the 4th and 7th nucleotides.

### The bull sperm miRnome

#### Overall description

**Fig. 5:**
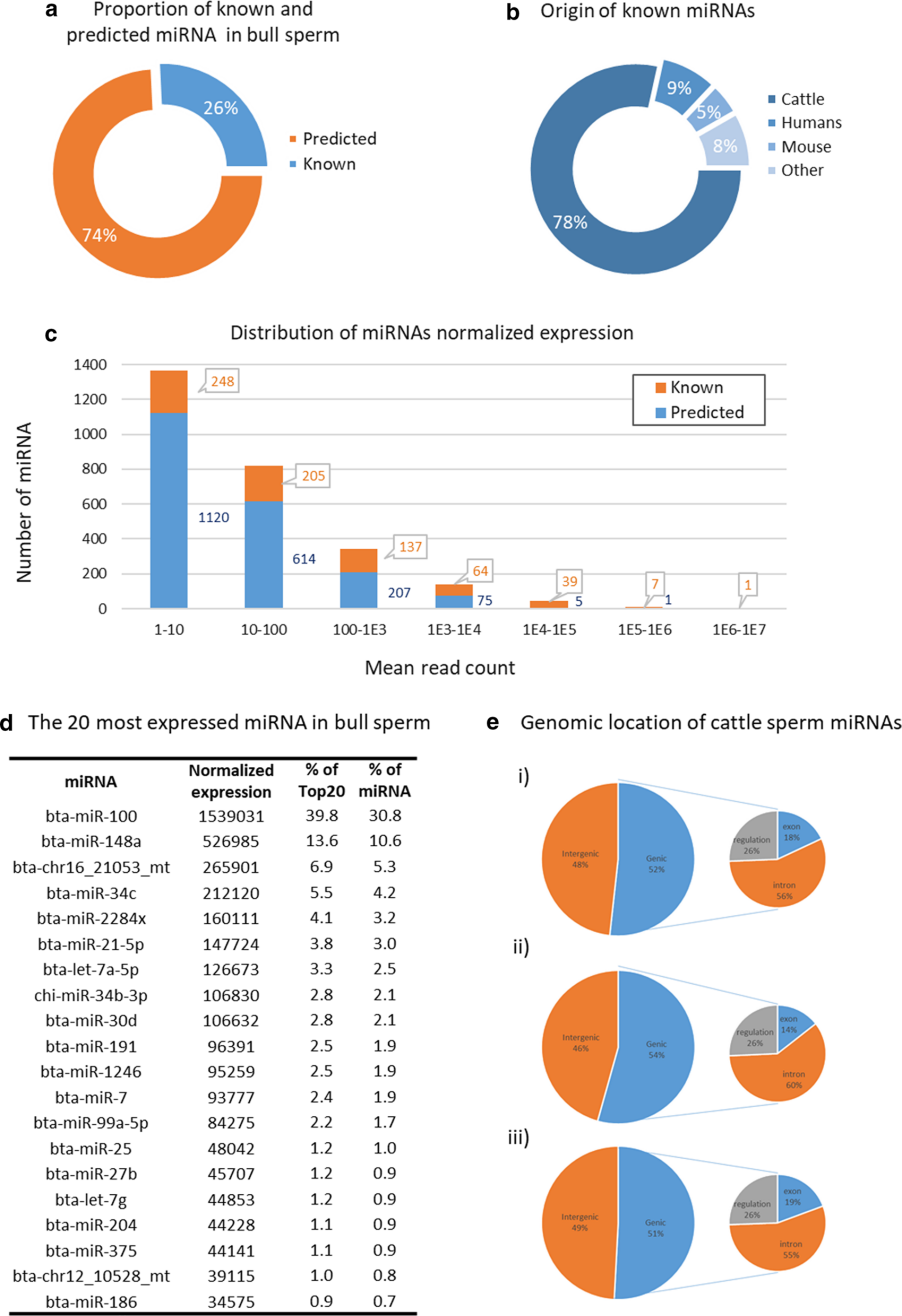
Bull sperm miRNA content. **a** Using miRDeep2 software, 2723 miRNAs were identified in bull sperm, including 701 (26%) known and 2022 (74%) predicted miRNAs. **b** Among known miRNAs, 550 were already described in cattle, 60 in human, 33 in mouse and 58 in other species. **c** The vast majority (80%) of miRNAs were expressed at low levels (2187 miRNAs expressed below 100 mapped reads on average), and only 53 miRNAs were highly expressed (above 10,000 mapped reads). **d** Twenty miRNAs account for 75% of the total miRNA mapped reads, including bta-mir-100, which alone accounts for 30% of miRNA expression (40% of the mapped reads for the top twenty miRNAs). **(e)** miRNA location within several genomic features, including intergenic or genic regions (introns, exons, and other regulatory regions, such as promoter, TSS, TTS, 3p or 5p UTR). Percentages shown on each rightmost “Pie of Pie” chart are expressed relative to the genic miRNAs. (i) Global distribution of sperm miRNAs. (ii) Distribution of known mRNAs; (iii) Distribution of predicted miRNAs

Based on miRDeep2 software, 2723 unique miRNAs were detected, belonging to 2579 miRNA precursors (pre-miRNAs). Among them, 701 (26%) were already known (550 in cattle, 60 in humans, 33 in mouse, and 58 in other species), corresponding to 635 known cattle pre-miRNAs (Fig. 5a, b). The remaining 2022 predicted miRNAs were associated with 1944 predicted pre-miRNAs (Additional file 3: Table S1). A total of 275 pre-miRNAs, including 184 known pre-miRNAs, were covered by both 5p and 3p miRNAs, while 26 predicted miRNAs may be produced by 97 pre-miRNA belonging to miRNA families (ranging from two to seven members). As shown by the distribution of normalized expression (Fig. 5c and Additional file 3: Table S7), the vast majority (80%) of miRNAs (6% of the known and 85% of the predicted miRNAs) were expressed at low levels (2187 miRNAs expressed with fewer than 100 mapped reads on average) and only 53 miRNAs were highly expressed (more than 10,000 mapped reads). The most highly expressed miRNA, bta-mir-100, exhibited about 1.5 M mapped reads.

The 20 most expressed miRNAs (> 35,000 mapped reads) accounted for 77% of all miRNA-associated reads (Fig. 5d). Bta-mir-100 accounted for 40% of the mapped reads for these top twenty, and for one-third of all miRNA reads. The second most expressed miRNA (bta-mir-148) was three times less expressed, accounting for 13.6% of the mapped reads from the top twenty. Interestingly, these top twenty miRNAs included 18 known and 2 putative miRNAs.

### Genomic distribution and correlation of expression

The half of miRNAs (52%) are located within genic features (10 kb interval), including within introns (29%), exons (9%), or other regulatory regions such as the promoter, TSS, TTS, 3p or 5p UTR (13%). As illustrated in Fig. 5e, known and predicted miRNAs showed nearly the same distribution, with known miRNAs being a little bit more frequently located within genic regions (54 vs 51% for predicted miRNAs). Based on miRBase standards (10 kb windows), 216 miRNA genomic clusters could be defined along the bovine genome (Additional file 3: Table S8), containing 532 miRNAs (i.e., 20% of total sperm miRNAs).

**Fig. 6:**
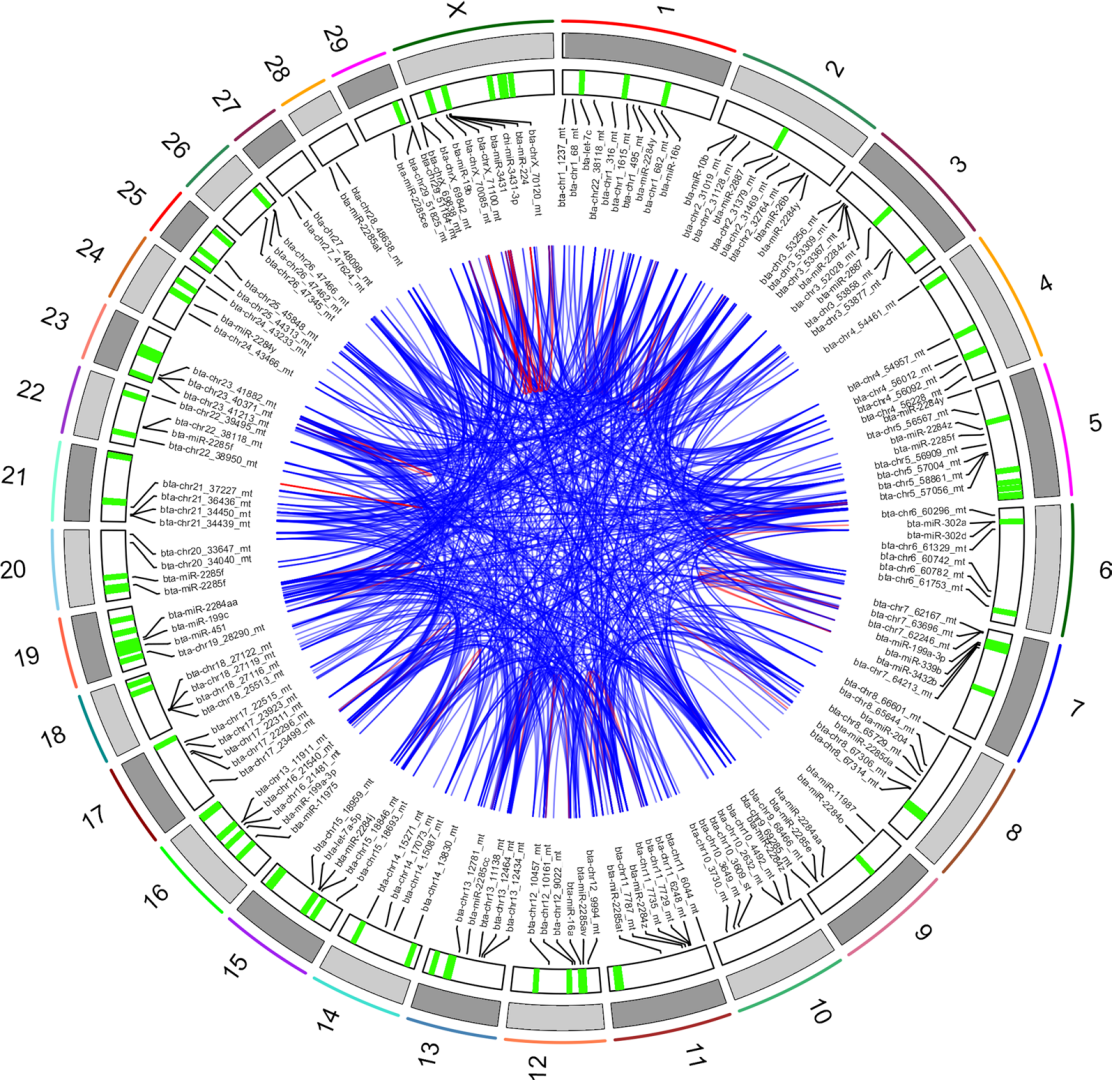
Circos plot depicting correlations between miRNAs. Chromosomes are indicated on the first outer track. Genomic clusters are indicated on the second track by green lines. All significant correlations (|r| > 0.9, adjusted p-value < 0.00001) were visualized, altogether involving 283 miRNAs. A subset of these miRNA is shown on the third track. Correlations between miRNA pairs are shown in the center of the plot, using blue and red lines for positive and negative correlations, respectively

To highlight genomically clustered miRNAs that could in fact be co-expressed on the same primary transcript, expression correlations were calculated between each pair of the top 1580 miRNAs with mean expression level greater than 10 in at least one breed. While a majority (66%) of the 1.2 million correlation coefficients clustered near zero, significant correlations were observed and are reported in Additional file 3: Table S9. Only 45 significant strongly negative correlations (*r* < − 0.7 and adjusted *p* value < 0.001) involving 59 miRNAs were observed, compared to 3357 significant strongly positive correlations (*r* > 0.75 and adjusted *p*-value < 0.001) involving 755 miRNAs. As illustrated by the Circos plot (Fig. 6), most correlations involved miRNA located on separate chromosomes.

Correlations between genomically clustered miRNAs showed a distinct distribution (Additional file 3: Table S10 and Additional file 9: Fig S8), suggesting stronger correlations than for non-clustered miRNAs. However, only 77 out of 216 (about one-third) of the genomic clusters showed strong expression correlation between miRNAs. In particular, Cluster 170 had 10 strong correlations between 12 miRNAs (bta-miRNA-381, bta-miRNA-382, bta-miRNA-134, mmu-miRNA-409-3p, bta-miRNA-409a, bta-miRNA-369-3p, bta-miRNA-410, bta-miRNA-3956, hsa-miRNA-382-3p, bta-miRNA-323b-3p, bta-miRNA-656, bta-miRNA-369-5p) among the 19 miRNAs located in this 22 kb cluster. Likewise, clusters 1, 3, 5 and 7 showed strong correlations between at least 4 miRNAs (bta-miRNA-411a, bta-miRNA-323, bta-miRNA-543, bta-miRNA-495, bta-miRNA-411b, hsa-miRNA-329-3p for Cluster 1; bta-miRNA-431, bta-miRNA-127, bta-miRNA-432, mmu-miRNA-127-5p for Cluster 3; bta-miRNA-19a, bta-miRNA-20a, bta-miRNA-19b, bta-miRNA-18a, bta-miRNA-17-5p for Cluster 5; and bta-miRNA-302b, hsa-miRNA-302a-5p, bta-miRNA-302a, bta-mir-302d for Cluster 7).

When considering correlations above |0.5|, 43 clusters of co-expressed miRNA could be defined, ranging from 2 to 16 co-expressed miRNAs (Table 2).

**Table 2:** Genomic clusters of miRNAs having strong expression correlations

Cluster number	Mean correlation	Clustered miRNA putatively co-expressed	Co-expressed in humans [45]
1	0.75	bta-mir-379 ~ bta-mir-411a ~ bta-mir-299 ~ bta-mir-380 ~ bta-mir-411b ~ bta-mir-323 ~ bta-mir-329b ~ bta-mir-543 ~ bta-mir-495 ~ bta-mir-154c	
2	0.72	bta-mir-532 ~ bta-mir-502a-1 ~ bta-mir-502a-2 ~ bta-mir-502b ~ bta-mir-500 ~ bta-mir-660 ~ bta-mir-502a-1	
3	0.80	bta-mir-431 ~ bta-mir-127 ~ bta-mir-432 ~ bta-mir-136	
4	0.72	bta-mir-450b ~ bta-mir-450a-1 ~ bta-mir-450a-2	Yes
5	0.91	bta-mir-17 ~ bta-mir-18a ~ bta-mir-19a ~ bta-mir-20a ~ bta-mir-19b	Yes
6	0.60	bta-mir-363 ~ bta-mir-19b-2 ~ bta-mir-20b ~ bta-mir-18b ~ bta-mir-106a	Yes
7	0.89	bta-mir-302b ~ bta-mir-302a ~ bta-mir-302d	Yes
8	0.64	bta-mir-2443 ~ bta-let-7b	Yes
10	0.65	bta-mir-10174 ~ bta-mir-27b ~ bta-mir-24-1	
14	0.74	bta-mir-429 ~ bta-mir-200a ~ bta-mir-200b	Yes
15	0.75	bta-mir-99b ~ bta-let-7e ~ bta-mir-125a	Yes
16	0.76	bta-mir-449c ~ bta-mir-449b ~ bta-mir-449a	
18	0.85	bta-mir-93 ~ bta-mir-25	Yes
21	0.76	bta-mir-182 ~ bta-mir-96 ~ bta-mir-183	Yes
22	0.68	bta-mir-24-2 ~ bta-mir-27a	Yes
23	0.75	bta-let-7a-1 ~ bta-let-7f-1 ~ bta-let-7d	
24	0.91	bta-mir-3431 ~ bta-mir-224	
25	0.67	bta-mir-105a ~ bta-mir-767 ~ bta-mir-105b	
29	0.65	bta-mir-16b ~ bta-mir-15b	Yes
30	0.70	bta-mir-181b-2 ~ bta-mir-181a-2 ~ bta-mir-181a-2 ~ bta-mir-181b-2	Yes
34	0.74	bta-mir-16a ~ bta-mir-15a	Yes
41	0.89	bta-mir-34b ~ bta-mir-34c	
47	0.75	bta-mir-29b-2 ~ bta-mir-29c	Yes
49	0.70	bta-mir-181b-1 ~ bta-mir-181a-1	
56	0.80	bta-mir-132 ~ bta-mir-212	
57	0.60	bta-mir-195 ~ bta-mir-497	Yes
61	0.61	bta-mir-2904-1 ~ bta-mir-2887-1	
70	0.64	bta-mir-7857-1 ~ bta-mir-7857-2	
72	0.55	bta-mir-1-2 ~ bta-mir-133a-2	
80	0.68	bta-mir-29a ~ bta-mir-29b-1	Yes
83	0.73	bta-mir-141 ~ bta-mir-200c	No
91	0.50	bta-mir-374b ~ bta-mir-421	
93	0.91	bta-mir-222 ~ bta-mir-221	No
111	0.65	bta-chr7_62503 ~ bta-chr7_62509	
118	0.89	bta-chr9_69352 ~ bta-chr9_69353	
125	0.78	bta-chr11_8402 ~ bta-chr11_8403	
140	0.65	bta-chr14_15810 ~ bta-chr14_15827	
145	0.99	bta-chr16_21540 ~ bta-chr16_21541	
152	0.65	bta-chr17_25055 ~ bta-chr17_25086	
169	0.51	bta-mir-6522 ~ bta-chr21_37502	
170	0.72	bta-mir-1185 ~ bta-mir-3956 ~ bta-mir-381 ~ bta-mir-487b ~ bta-mir-411c ~ bta-mir-487a ~ bta-mir-382 ~ bta-mir-134 ~ bta-mir-154a ~ bta-mir-154b ~ bta-mir-409a ~ bta-mir-412 ~ bta-mir-369 ~ bta-mir-410 ~ bta-mir-323b ~ bta-mir-656	
183	0.78	bta-chr23_40757 ~ bta-chr23_42270	
211	0.78	bta-mir-6526-3 ~ bta-chrX_70042 ~ bta-chrX_70050 ~ bta-chrX_70048 ~ bta-chrX_70052 ~ bta-chrX_70054	

Additional file 3: Tables S8 and S10 for detailed information. Empty cells correspond to none observed cluster in human article

Intriguingly, most clusters gather either known or putative miRNAs, with the exception of Clusters 169 and 211, which comprise both known and predicted miRNAs.

**Fig. 7:**
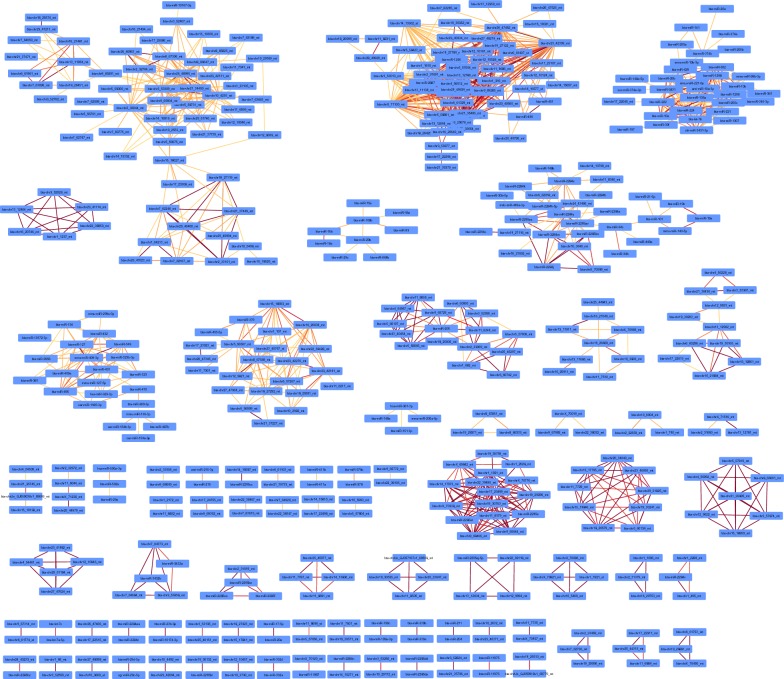
Correlation networks of positively correlated miRNAs. Correlation networks of miRNA pairs with significant positive correlations above 0.9 as drawn by Cytoscape. Edges are colored according to the correlation level (0.9 in yellow to 1 in red)

Regarding significant correlations between miRNAs that are not genomically clustered, we identified 77 correlation networks made up of 2 to 61 positively correlated miRNAs (Fig. 7).

Interestingly, five putative functional regulation networks were identified based on their correlation pattern (for instance the positive correlation between bta-miR-24-3p and bta-miR-107, while bta-miR-24-3p and bta-miR-107 are negatively correlated with bta-chr7-64527_mt suggests that bta-miR-24-3p may regulate bta-chr7-64527_mt, which may in turn regulate bta-miR-107, see Additional file 10: Fig S9).

**Fig. 8:**
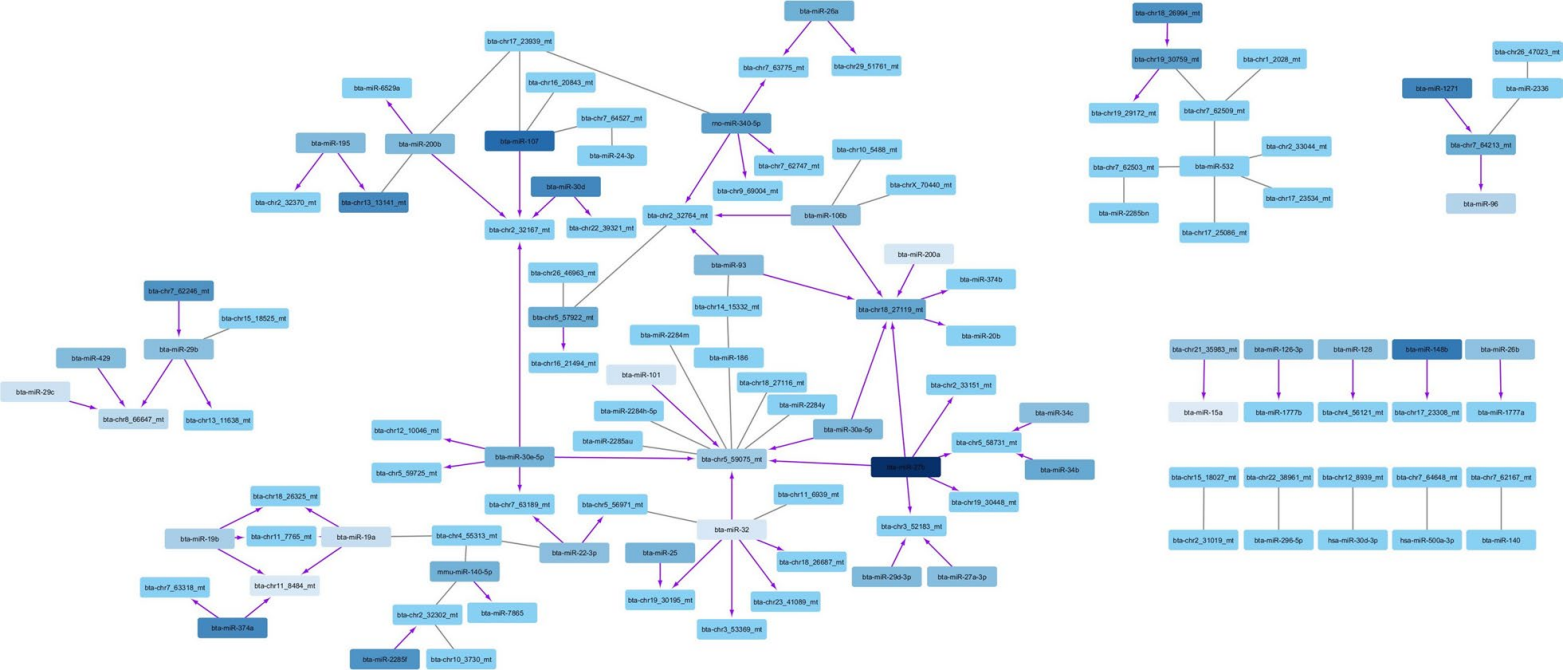
Correlation networks of negatively correlated miRNAs. Partially oriented correlation networks of miRNA pairs with significant negative correlations less than − 0.7 (any pair) or less than − 0.5 (any miRNA paired with a miRNA located within its target genes), as drawn by Cytoscape. Nodes are colored according to the mean correlation level (from − 0.5 (light blue) to − 0.9 (dark blue)). Purple arrows indicate correlated pairs made up of a given miRNA (arrow start) and another miRNA located within a target gene of the first (arrow end). Grey edges without arrows indicate that linked miRNA are correlated, without any known functional interaction

Likewise, negatively correlated miRNAs defined 14 correlation networks, ranging from 2 to 76 miRNAs (Fig. 8). Interestingly, among these, 41 “master” miRNAs were found to be negatively correlated with 46 master miRNAs, thus defining putative regulatory networks with their host genes.

### The repertoire isomiRs in bull sperm

**Fig. 9:**
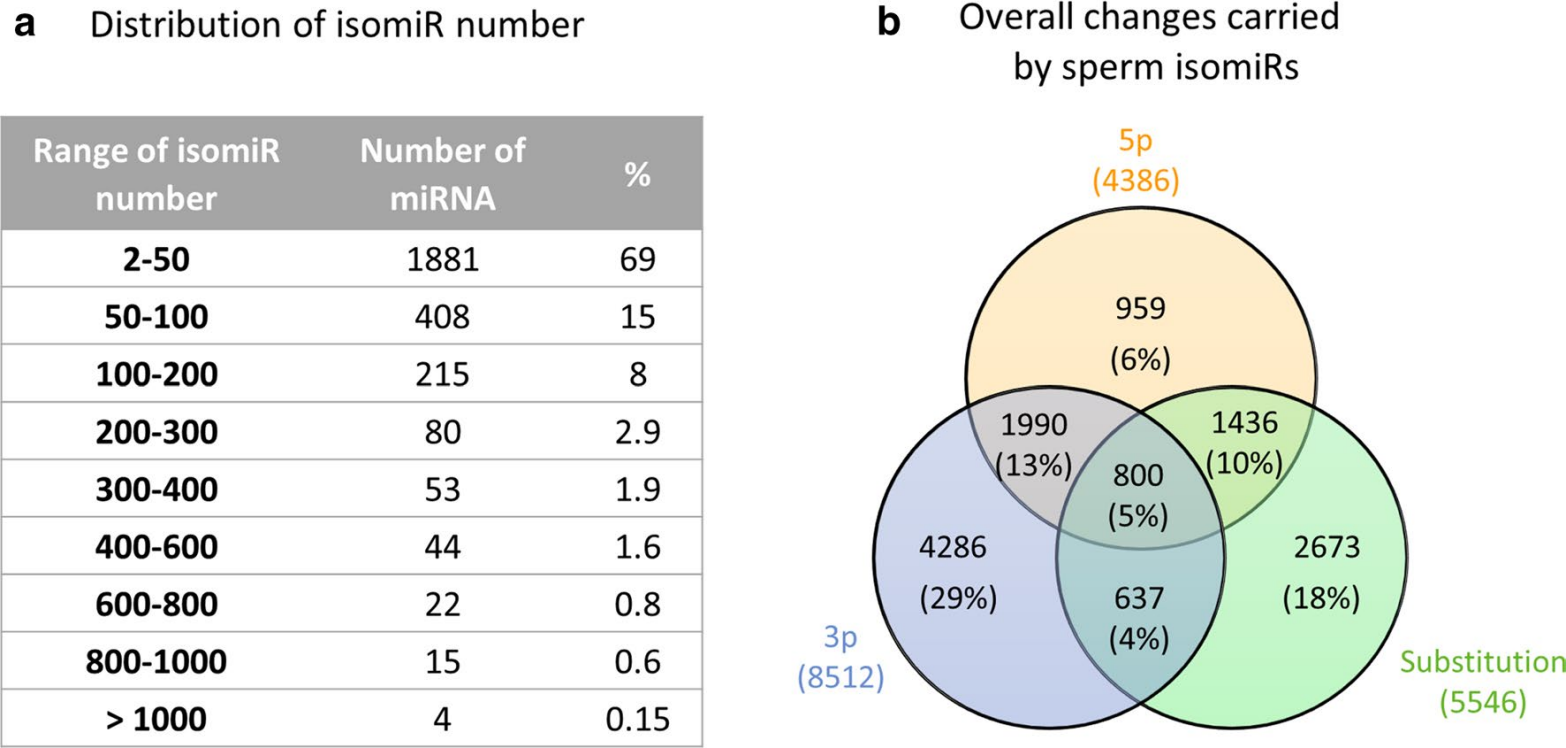
The diversity of isomiRs in bull sperm. **(a)** 2722 out of 2723 sperm miRNA were represented by at least 2 isomiRs, with a vast majority (1882, i.e., 69%) represented by fewer than 50 isomiRs. Only 4 miRNAs (0.15%) exhibited more than 1000 isomiRs. **(b)** The number and percentage of isomiRs carrying 5p or 3p changes, a substitution, or a combination of these changes

We also studied the diversity of isomiRs, i.e., changes in the mature miRNA sequence, including substitutions (Polymorphic isomiRs), cleavage variations (5p and 3p isomiRs), and additions at either the 5p or 3p end (see Additional file 11: Fig S10 for some examples of isomiRs and their nomenclature). Altogether, 192,895 isomiRs were identified among the 2723 sperm miRNA (Additional file 3: Table S11). The number of isomiRs per miRNA varied greatly, ranging from 1 (bta-chr18_27604_mt) to 1439 (chi-miRNA-34b-3p), with on average 71 ± 126 isomiRs per miRNA. As shown in Fig. 9a, the majority (1882, i.e., 69%) of miRNAs were represented by fewer than 50 isomiRs, and only 4 miRNAs (0.15%) exhibited more than 1000 isomiRs. No correlation between the number of isomiRs and the expression level of miRNAs was observed.

In agreement with the levels of miRNA expression, most isomiRs were found to be expressed at low levels (Additional file 3: Table S12): only 2329 isomiRs (1.2%) exceeded a mean normalized expression of 100 mapped reads in at least one breed, and about 35,785 isomiRs (20%) showed expression levels between 1 and 100 reads. The vast majority of isomiRs accounted for only a small proportion of miRNA expression: 167,776 isomiRs (87%) contributed less than 1% to total miRNA expression, while only 2916 isomiRs (3%) from 2445 miRNAs contributed more than 25% of total miRNA expression. Of note, 101 canonical sequences described in miRBase (14% of known expressed miRNAs) were not detected at all in our data. In addition, the defined canonical sequence was found to be the most highly expressed for only 1269 miRNAs (47%), while the most expressed isomiR differed from either the canonical miRNA published in miRBase or the predicted canonical sequence identified by mirDeep2 in 75% and 46% of cases, respectively. For instance, the published canonical form of bta-miRNA-100 (AACCCGTAGATCCGAACTTGTG) accounted for only 10% of the miRNA read count, while bta-miRNA-100 isomiR AACCCGTAGATCCGAACTTGT (deletion of G in 3p) accounted for 30%. Overall, on average the most expressed sequences were found to account for 51% of total miRNA expression, ranging from 7 to 100%. The 20 most expressed isomiRs accounted for 49% of the total isomiR expression and are related to 10 miRNAs, including bta-miRNA-100 (6 isomiRs in the top twenty), the most abundant miRNA in bull sperm cells. On average, isomiRs accounted for 4.5% of the expression for each miRNA, ranging from 0.06 to 100%.

To avoid bias due to sequencing errors (0.1% of sequencing errors may produce up to 30,000 isomiR artifacts), we focused on 14,765 isomiRs accounting for more than 1% of each miRNA’s expression and expressed in at least half of the animals of at least one breed (Additional file 3: Table S13). Production of multiple isoforms appeared to be a general mechanism, as only 1984 sequences were identified as the canonical miRNAs: 3p, 5p and substitutions within the canonical sequence were detected for 81%, 57% and 77% of miRNAs, respectively. Altogether, 58% and 30% of isomiRs showed changes at their 3p and 5p ends, respectively, while 38% exhibited at least one substitution within the canonical sequence. Single 3p changes and substitutions were the most frequent variations (29% and 18% of isomiRs, respectively). About 5% of isomiRs were found to carry both 5p and 3p changes as well as substitutions (Fig. 9b).

**Fig. 10:**
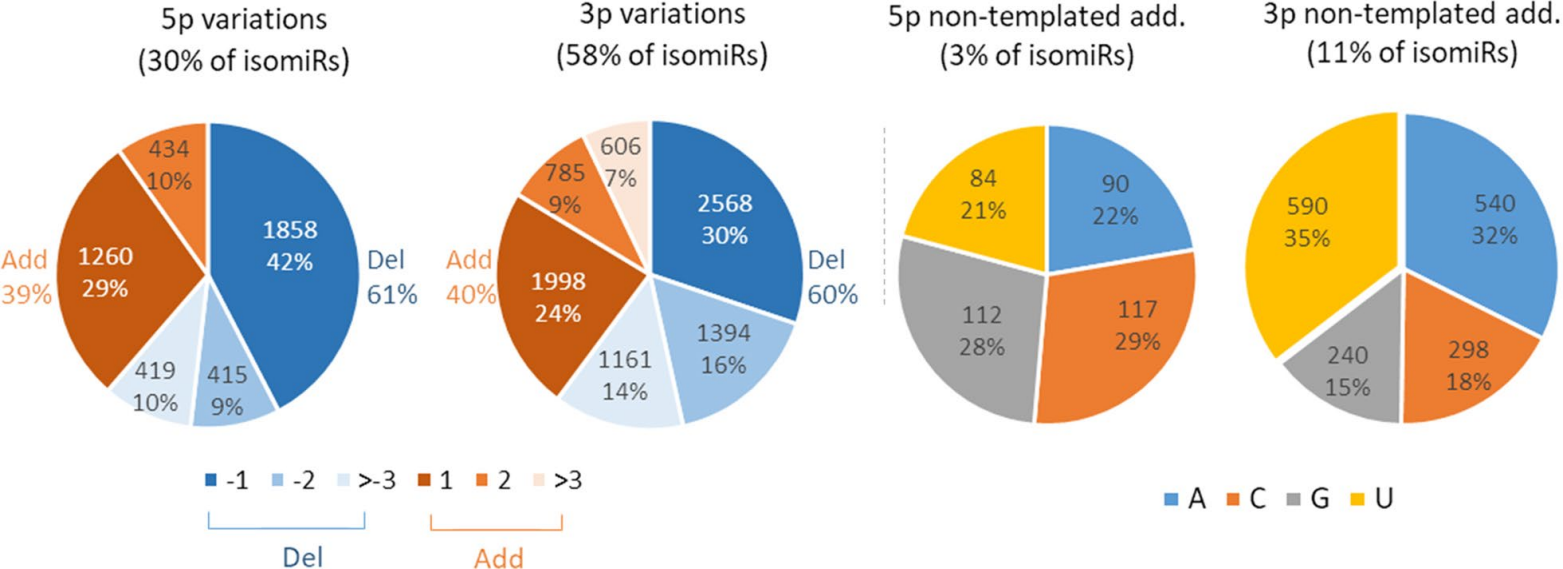
Frequency of end variations affecting isomiR sequences. Percentages of addition and deletion at either 5p or 3p ends were computed based on unique sequence counts. Deletions were the most frequent variations, accounting for ~ 60% of variations affecting 5p and 3p ends. Single nucleotide changes were likewise the most frequent variation (71% and 54% of 5p and 3p changes). Regarding non-templated additions, additions of Cytidine (C) or Guanosine (G) were frequently observed at 5p (29% and 28%, respectively), whereas additions of Adenosine (A) and Uridine (U) were the most frequent 3p variations (32% and 35%, respectively)

As illustrated in Fig. 10, deletions accounted for ~ 60% of variations affecting 5p and 3p ends, with the addition or deletion of one nucleotide representing altogether 71% and 54% of 5p and 3p changes, respectively. Additions were usually consistent with the reference sequence, but 2071 isomiRs (14%) showed a non-templated nucleotide addition at either 5p (403) or 3p (1668) locations. Among them, only 86 substitutions (4%) may result from known genetic polymorphisms (Ensembl release 94). Addition of cytidine (C) or guanosine (G) were observed frequently at 5p (29% and 28%, respectively), whereas addition of adenosine (A) and uridine (U) were the most frequent 3p variations (32% and 35%, respectively).

**Fig. 11:**
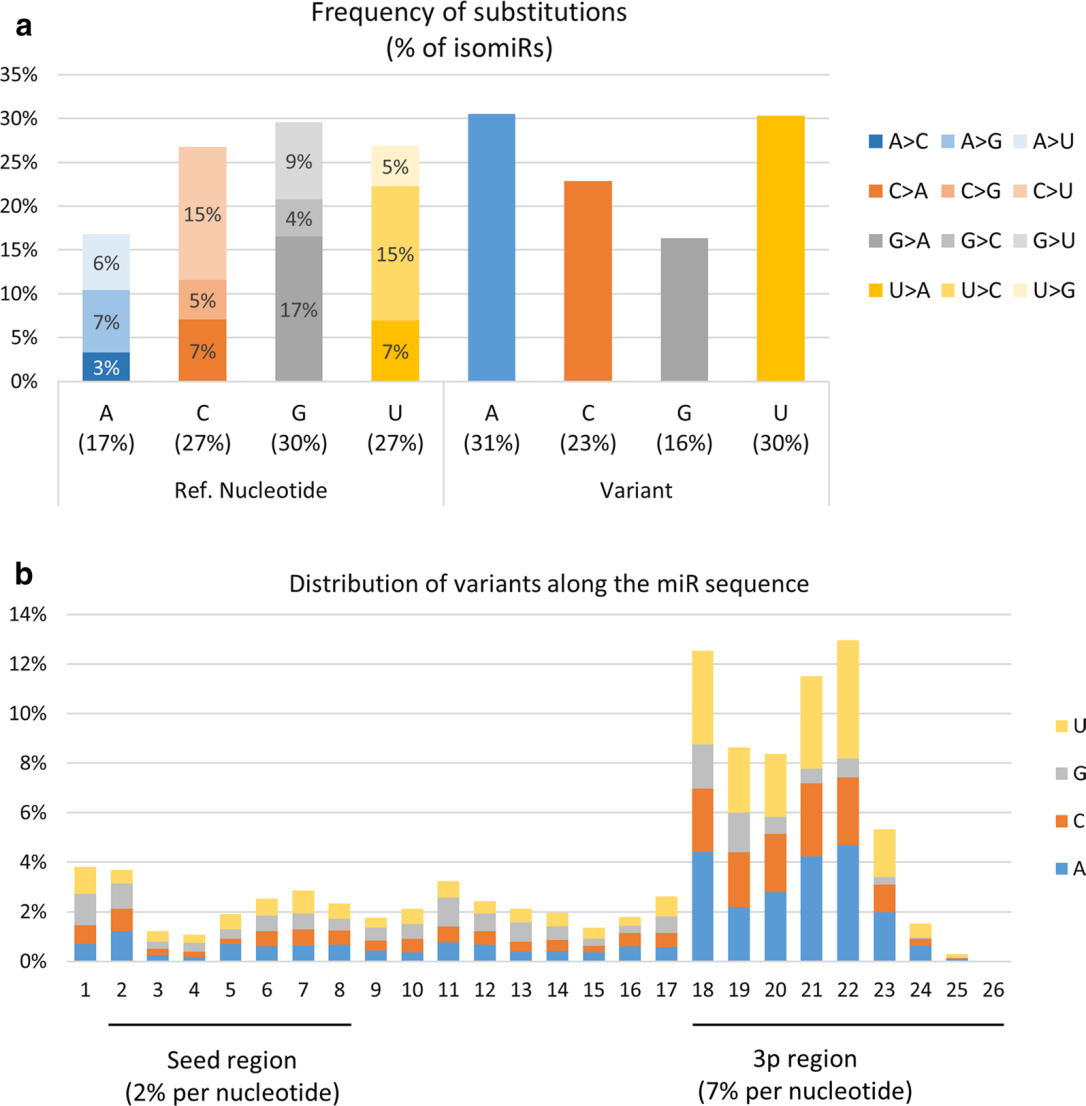
Frequency of substitutions affecting isomiR sequences. **a** Percentages of nucleotide substitutions were computed based on unique sequence counts. On average, G nucleotides were more frequently modified (30% of isomiR counts) than C/U (27%) and A (17%), being mainly replaced by A or U. Altogether, G > A, U > C and C > U substitutions were the most frequent polymorphisms, each accounting for ~ 15% of all polymorphisms. **b** The distribution of variations along the miRNA sequence clearly shows that substitutions are more likely to occur within the 3p region (61% of isomiRs, ~ 7% substitution per nucleotide) than the first 5p nucleotide (4%) or the seed region (16% of isomiRs, ~ 2% substitution per nucleotide). Substitutions at 5p were prone to be G or U nucleotides (33% and 28%, respectively), whereas substitutions to A or U nucleotides were observed more frequently at 3p (34% and 33%, respectively)

Regarding polymorphic isomiRs, about 6% of variations may be related to known genetic polymorphisms, the remaining 94% likely being produced by miRNA editing. As shown in Fig. 11a, G nucleotides were on average more frequently modified (30% of isomiR counts) than C/U (27%) or A (17%). However, when accounting for nucleotide usage within miRNAs (i.e., frequency of nucleotides within miRNA sequences), substitutions involving C and U appeared to be 50% and 10% more frequent than expected by chance. In contrast, G substitutions appeared 20% less frequently than expected (Additional file 12: Fig S11). Nucleotides were mainly replaced by A or U (Fig. 11a). Altogether, G>A, U>C and C>U substitutions were the most frequent polymorphisms, each accounting for ~ 15% of all polymorphisms.

The distribution of variations along the miRNA sequence (see Fig. 11b) clearly showed that substitutions are more likely to occur within the 3p region (61% of isomiRs, ~ 7% substitution per nucleotide) than the first 5p nucleotide (4%) or the seed region (16% of isomiRs, ~ 2% substitution per nucleotide). Distinct patterns of variation were observed when comparing 5p and 3p regions: substitutions at 5p were prone to be G or U nucleotides (33% and 28%, respectively), whereas substitutions to A or U nucleotides were observed more frequently at 3p (34% and 33%, respectively).

### Diversity of sncRNAs across breeds

**Fig. 12:**
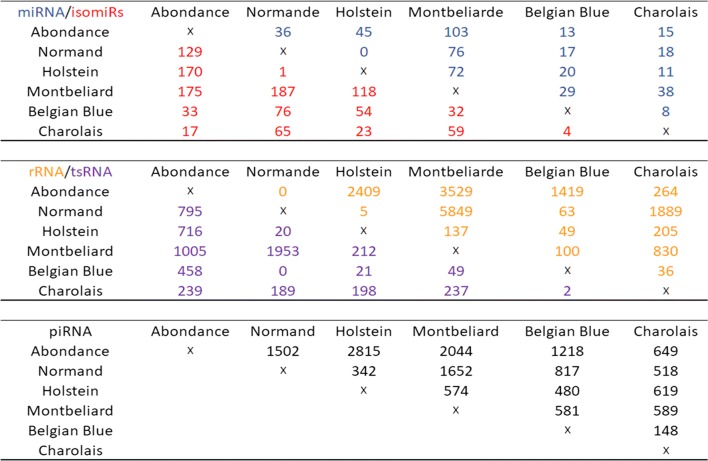
Differential expression of sncRNAs across breeds. Number of sncRNA per class showing significant differential expression between pairs of breeds (adjusted *p*-value < 0.05). Many sequences were found to be differentially expressed between at least two breeds, whatever the sncRNA class. Interestingly, the Abondance and Monbeliarde breeds showed the highest number of differentially expressed sncRNAs, whatever the class and the breed comparison. In contrast, the Belgian Blue vs Charolais as well as the Holstein vs Normand comparisons yielded the lowest number of differentially expressed sncRNAs

Differential expression analysis was used to explore the expression patterns of sncRNAs (miR, piRNA, tRNA and rRNA) across breeds (Additional file 3: Table S14). Figure 12 provides, for each sncRNA class, the number of differentially expressed features (adjusted *p*-value < 0.05) observed for each comparison of breeds.

Many sequences were found to be differentially expressed in each pairwise breed comparison, regardless of the sncRNA class. The Monbeliarde breed showed the highest number of differentially expressed sncRNAs, whatever the class and breed comparison. For instance, 63 miRNAs and 2089 rRNAs were found to be differentially expressed on average when considering comparisons between the Montbéliarde and other breeds, whereas only 22 and 720 differentially expressed miRNAs and rRNAs, respectively, were identified on average for other comparisons. In contrast, the Belgian Blue *vs* Charolais as well as the Holstein *vs* Normand comparisons yielded the smallest number of differentially expressed sncRNAs, whatever the class. For instance, no differentially expressed miRNAs and only one differentially expressed isomiR could be identified between Holstein and Normand. Likewise, only a few differential sequences were identified between Belgian Blue and Charolais, with only 8 miRNAs and 4 isomiRs (compared to 15 miRNAs and 76 isomiRs on average). A subset of five miRNA was chosen to perform a biological validation by qRT-PCR: two miRNA (bta-chr4_54509 and bta-mir-148b) and two isomiRs (bta-isomir-26a-1_1 and bta-isomir-26a-1_2) differentially expressed between Abondance and Normande breeds, as well as one miRNA differentially expressed between Charolais and Normande (bta-chr16_21053). Five additional bulls, which were not included in the RNA-Seq study, were chosen for each breed. On these five miRNA, three confirm statistically the RNA-Seq results, one showed the same trend but failed to produce a statistically significant result and no differential expression was observed for the last one (Additional file 3: Table S15). Altogether, NGS results were confirmed in 80% of cases (60% with statistical significance).

**Fig. 13:**
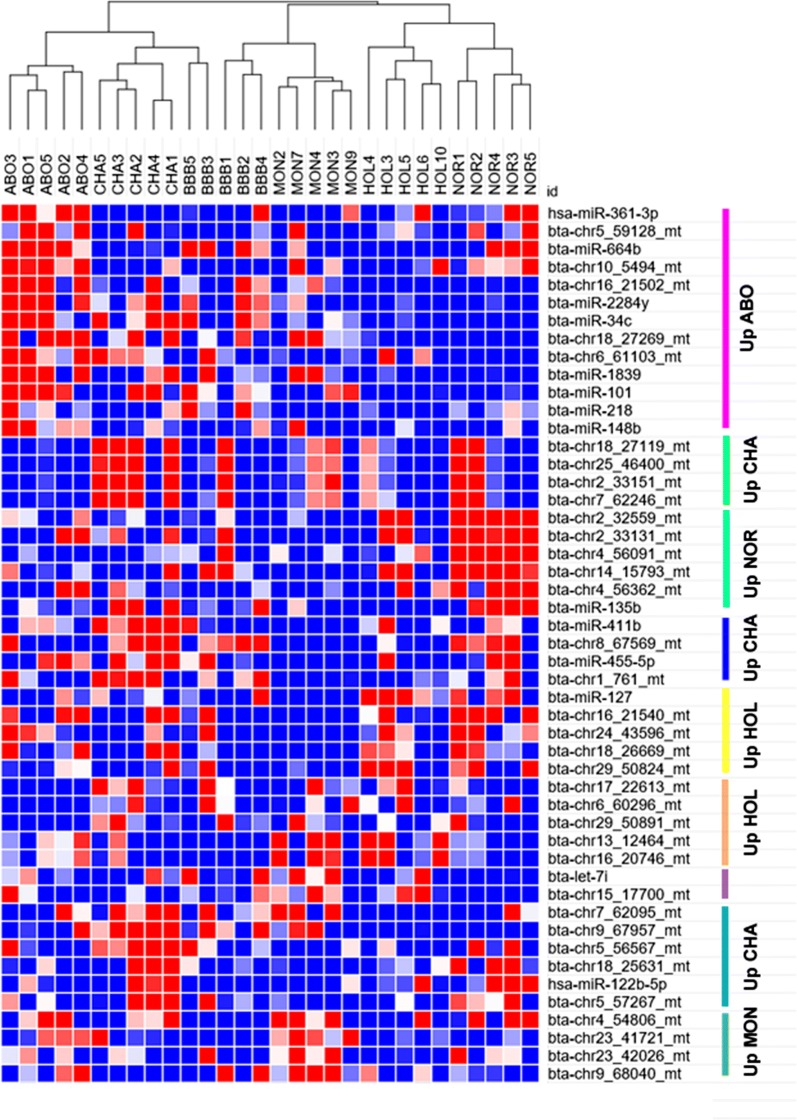
Hierarchical clustering based on miRNA expression. Breeds could be distinguished based on 294 differentially expressed miRNA, among which 50 are depicted in the diagram. Breed specific expression patterns could be observed, some miRNA being upregulated in one or a few breeds

Consistent with the observed differential expression, breeds could be distinguished by hierarchical clustering on miRNA, with Holstein and Normande being clustered close together, while in contrast the Abondance and Normande breed belong to the most distant clusters (Fig. 13). The Belgian Blue breed appeared to be more diverse, with 2 bulls close to the Charolais breed and 3 bulls close to the Monbeliarde breed.

**Fig. 14:**
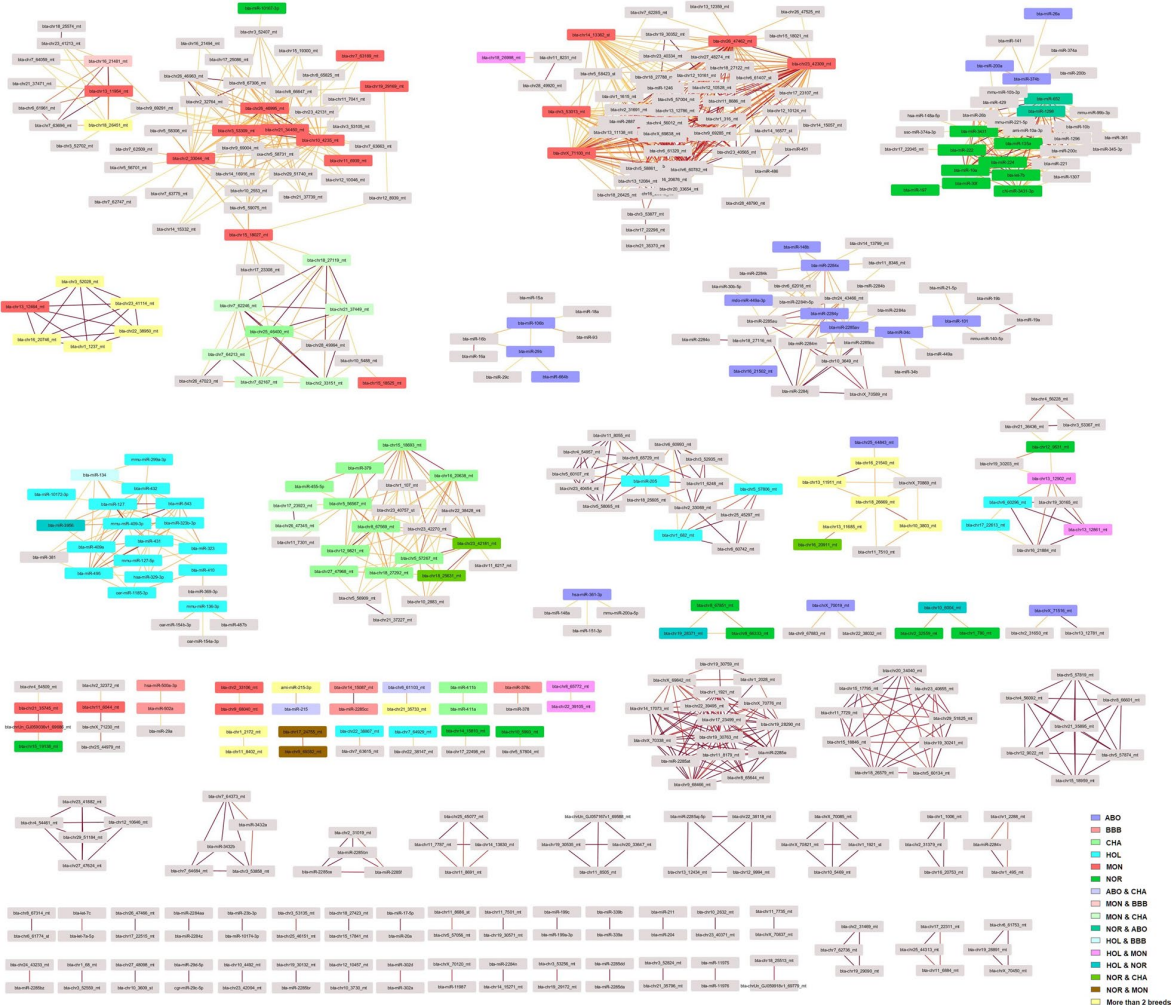
Correlation networks of positively correlated miRNAs. Correlation networks of miRNA pairs with significant positive correlations above 0.9 (any pair) or above 0.75 (pair including a miRNA exhibiting differential expression across breed), as drawn by Cytoscape. Nodes were colored according to the miRNA-specific expression across breeds (i.e., up-regulation in one or a few breeds compared to the others)

Noteworthy, breed-specific expression patterns could be observed, with several groups of miRNA being differentially expressed between one (or a few) breed and the others. Altogether, about half of differentially expressed miRNA could be associated to such specific patterns. Interestingly, these miRNAs were found to cluster in the previously described correlation networks (Fig. 7), with miRNA having a similar pattern clustering within the same correlation networks, as illustrated by the node color code in each network (Fig. 14). Thus positive correlations between miRNA are, at least in part, indicative of coordinated expression in each breed.

Putative targets of differentially expressed known miRNAs were identified using Targetscan 7.2 and filtered on “weighted context ++ score percentile” (≥ 95%), resulting in a set of 2826 targets used for a gene ontology analysis. Several enriched biological terms were identified, including animal organ morphogenesis, cell development, anatomical structure morphogenesis, and regulation of gene expression. Surprisingly, by focusing on the two most highly enriched terms (organ morphogenesis and cell development), 71% of the annotated genes with significantly differential expression among breeds were characterized by highly specific expression in the Montbéliarde breed compared to another. Moreover, the majority of miRNAs targeting these same genes were found to be under-expressed in the Montbéliarde sperm cells (about 90% of the cases).

**Fig. 15:**
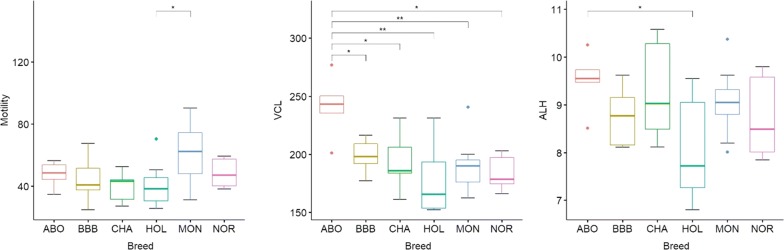
Distribution of motility related sperm quality parameters across breeds. Boxplots illustrate the distribution of three parameters (progressive motility (%), curvilinear velocity (VCL, μm/s) and amplitude of lateral head displacement (ALH, μm)), showing the minimum, first quartile, median, third quartile, and maximum for each breed. Significant pairwise comparison (Wilcoxon rank sum tests) are depicted above boxplots (*p value under 5%, **p value under 5‰)

Noteworthy, semen quality parameters were shown to vary across breeds, especially parameters related to motility such as curvilinear velocity (VCL, μm/s), amplitude of lateral head displacement (ALH, μm) and progressive motility (%) (Fig. 15 and Additional file 3: Table S16).

Since a previous study reported differentially expressed miRNA and piRNA between high and low motile spermatozoa [30], association between quality parameters and breed differentially expressed miRNA and piRNA was assessed. Firstly, among the 196 differentially expressed sequences described in Capra et al. [30], 156 were also identified in our dataset, corresponding to 121 different miRNA. On these, 22 (18%) were also differentially expressed between breeds (Additional file 3: Table S17). Then correlations were computed between quality parameters and miRNA/piRNA expression level in each breed (Additional file 3: Table S18). With only five sample per breed, it was not possible to take inter-individual variation into account and evaluate whether variation among breeds is larger than individual variation within each breed. Thus, the mean expression level was computed per breed for each miRNA/piRNA and correlations were estimated between the mean expression level and the mean of each functional parameter. When considering parameters related to motility (VCL, ALH, motility), 833 miRNA were found to be correlated to either VCL, ALH, motility, 129 being also differentially expressed between breeds and 91 being differentially expressed in Abondance breed, which shows significant difference in VHL and ALH parameters compared to the other breeds. In addition, 38 miRNA belong to miRNA differentially expressed between High and Low motile spermatozoa identified by Capra et al. [30]. Likewise, 5464 piRNAs were found to be correlated to motility related parameters, 259 being differentially expressed between breeds and 250 being differentially expressed in Abondance breed. These piRNA belong to all clusters associated to High motile (64 clusters) and Low motile spermatozoa (15 clusters) identified by Capra et al. [30].

## Discussion

The study of sperm RNAs is challenging, as spermatozoa harbor low amounts of highly fragmented RNA and are to some extent resistant to lysis using conventional methods. As a consequence, numerous procedures have been developed, often leading to highly variable RNA content and making it difficult to compare between samples or studies, especially across species. In our hands, the published protocols led to partial lysis of cattle sperm (evaluated by microscopic examination) and low RNA yield. In contrast, we were able to reproducibly apply our novel protocol to 40 sperm samples. On average, about 60 ng of total RNA can be recovered from about 30 million spermatozoa (2 straws), i.e., about 2 fg on average per frozen spermatozoa. This is consistent with the published estimate of 5 fg/sperm in swine [46], 2–20 fg/sperm in stallions [47], and 5–15 fg/sperm in humans [9, 48]. In agreement with previous studies, no 18S and 28S rRNA peaks could be detected by electrophoretic assay, indicative of low contamination by somatic cells [49]. Most importantly, the conditions for RNA extraction and sequencing enabled us to establish a comprehensive repertoire of cattle sperm-borne sncRNA, which could serve as reference for later studies focusing on sperm quality characteristics and fertility.

### The overall small RNA content of bull sperm

Consistent with Spermbase data on human and mouse [19], the most abundant classes of sncRNA in bull sperm were rRNAs, piRNAs, miRNAs and tsRNAs, with rRNA being the most highly represented class. However, this finding contrasts with Spermbase data, where tsRNA account for more reads than any other sncRNAs. Our findings also agree with previous reports suggesting that piRNAs are highly abundant sncRNAs in sperm [57].

Reads annotated as mRNA (0.4% of reads) represented degraded transcript fragments from 3250 genes, of which a substantial portion is involved in spermatogenesis. This information supports the hypothesis that these mRNA fragments are remnants from earlier stages of spermatogenesis rather than derived from contaminating somatic cells in our preparation.

Though no 18S and 28S rRNA peaks could be detected by electrophoretic assay, about 25% of reads were annotated as rRNAs, mainly 18S (29%) and 28S (23%) rRNAs. However, consistent with a previous report in humans, they appeared to be fragmented, with particular patterns of peaks and read-poor regions, suggesting that this degradation is achieved by a selective RNA cleavage [20]. Of note, mature spermatozoa are thought to be depleted in rRNAs, due at least in part to the expulsion of the cytoplasm as a droplet during maturation. The abundance of rRNA fragments in mature sperm suggests that expulsion through the cytoplasmic droplet is not complete and that cleavage may be of importance to avoid protein synthesis. Whether these rRNA fragments may also play a role at fertilization or during zygote development warrants future study.

Together with rRNAs, we identified piRNAs as a major constituent of sperm sncRNAs and observed a great diversity of piRNA-like sequences. In a previous study on cattle gonads, gametes, and embryos, Russel et al. [50] reported a distinct population of slightly shorter, 24–27 nt piRNA-like RNAs exhibiting many of the canonical characteristics of piRNAs but having weak or absent 1U bias. Consistent with these findings, piRNA-like RNAs identified in our study also appeared to be shorter, resulting mainly from a deletion at either ends. However, we still observed a 1U bias, although weaker than for piRNAs. No particular expression profile and no association with particular biogenesis classes could be observed. Thus, with the exception of their length, these piRNA-like RNAs showed all the same features of piRNAs and likely represent isoforms of piRNAs. Recently, different length variations of piRNAs have been discovered in several studies, and several exonucleases have been found to be required for piRNA end trimming in different species [51–53]. To our knowledge, no bioinformatic resource is yet available for the detection and annotation of piRNA isoforms, with the exception of IsopiRBank [54], which unfortunately does not cover the bovine species. However, consistent with statistics from IsopiRBank and general mechanisms of RNA editing, we found that length variations at the 3′ end were the most frequent changes affecting piRNA isoforms.

Besides their established role in repressing transposable elements and protecting the integrity of the genome in germ cells [55], growing evidence suggests that piRNAs also function in regulating protein-coding genes in germ cells and early embryo development [56] and play a role in sperm fertility [57]. A noteworthy result of our study was the overwhelming majority of reads annotated as intergenic-derived piRNAs, with a significant fourfold enrichment compared to the whole piRBase database. In addition, the sperm piRNAs were predominantly 29–31 nt in length; showed preference for uracil at their 5p terminus; had no adenine enrichment at nucleotide 10; and appeared to derive from transposons in very small number (only 5%). Altogether, this suggests that the piRNA population identified in bull mature sperm is mainly produced by the PIWIL1-directed production pathway rather than the PIWIL2/PIWIL4-directed secondary pathway in which piRNAs are typically produced from transposons or other repeat-associated regions. This is consistent with previous findings in mice, where studies showed that (i) in contrast to primordial testis, piRNAs are produced in adult testis independent of the ping-pong mechanism [58]; and (ii) cauda spermatozoa were enriched in piRNAs apparently derived from the primary PIWIL1-directed production pathway and possibly generated in situ in maturing spermatozoa [7]. Elucidating the role of bull sperm piRNA and piRNA-like in sperm fertility and early embryo development warrants further investigation.

Regarding tsRNAs, we identified 5p-tRHs as the predominant subgroup in bull sperm, in good agreement with results in human, mouse and rabbit sperm data [19]. Altogether, 5p-derived fragments represented 63% of all bull sperm tsRNAs, with tRF3s and 3p-tRHs being expressed at low levels (30× less expressed than 5p derived fragments). A similar pattern was also described in bull sera [59], but with a larger contrast, where 5p-derived fragments were 4000× more expressed than 3p ones. This discrepancy may be related to biological differences between tissues, as suggested by the extensive differences in tRF expression observed between human biofluids [60]. Alternatively, they may also reflect sperm changes in response to stress induced by the cryopreservation process. Indeed, tRHs are produced by specific cleavage in the anticodon-loop of mature tRNA by angiogenin under various cellular stress conditions, and changes in tRF relative expression have been described under pathological conditions [61]. In addition, oxidative stress has been shown to promote tRNA fragmentation [62], and altered 5p tRHs profiles have been observed in mouse sperm after a high-fat diet [16]. Whether tRFs expression profiles could be relevant biomarkers for sperm quality or bull management will require further work. Unlike many other studies, we identified i-tRFs as the second contributor (18%) to sperm tsRNAs, with more than 84% of these reads being produced by MT tRNAs, a disproportionately high contribution considering that there are only 22 MT tRNA sequences. Similar results have already been described in prostate cancer tumors, where i-tRFs derived from MT tRNAs are particularly abundant [63]. In our data, Gly and Glu isoacceptors were predominant in ejaculated bovine sperm cells, in good agreement with results obtained on cauda epididymis sperm cells in mice [64]. Interestingly, these isoacceptors have also been reported as over-represented in seminal plasma exosomes [65] and epididymosomes [64], which can transfer their tsRNA content to the sperm [66]. This epididymal cell–sperm communication mechanism has been proposed to link the paternal environment (i.e., nutrient availability) to the mature sperm, providing an adaptive metabolic advantage to the offspring [67]. Whether sperm tsRNAs derive from this mechanism or from the intrinsic ability of sperm to cleave tRNA is still unclear. Regarding the biological role of these sperm tsRNAs, several groups have provided compelling evidence in mice that they are involved in paternal epigenetic inheritance and early embryonic development through modification of embryonic gene expression [64, 68]. For instance, tRF5- Gly-CCC, -TCC, and –GCC were shown to repress MERVL, an endogenous retro-element active in the preimplantation embryo and regulating the expression of genes involved in totipotency and zygote genome activation [69]. Likewise, tRF- Gly-GCC and tRF-Glu-TTC were found to be downregulated in human sperm producing low-quality embryo in vitro [70]. Whether mis-regulation of some tsRNAs in sperm might contribute to poor sperm quality, abnormal early embryo development, and reduced fertility in cattle warrants future investigation.

### The bull sperm miRnome

Based on miRBAse, we identified 635 miRNAs, increasing by ~ 50% the known miRNA repertoire present in sperm. Indeed, 376 miRNAs have been identified in either human, mouse or rat sperm according to SpermBase [19], from which 216 have also been detected in the present study. Our results partly overlap those of previous studies on cattle sperm, both in terms of identified miRNAs and expression levels. For instance, 28 out of the 30 most expressed miRNA published by Stowe et al. [71] are also expressed at high levels in the present study, with the exception of bta-miRNA-199a-5p, which is weakly expressed. Likewise, expression levels of 404 shared miRNAs were also in good agreement with those measured by Capra et al. [30] (Additional file 3: Table S19).

Based on miRDeep2, 2022 additional miRNAs were predicted, associated with 1944 pre-miRNAs. Among these predicted miRNAs, 68 were also previously identified by Capra et al., and 5% were covered by both 5p and 3p miRNAs, increasing the likelihood of being true novel miRNAs. Of note, we report numerous known and predicted miRNA expressed at moderate and low levels. Our efficient extraction procedure, as well as the high sequencing depth in our study (35 M reads vs ~ 4.5 M per sample in previous studies), probably accounts for the wider panel of known and novel miRNAs detected in this study. Alternatively, discrepancies between studies may also be explained by different miRBase release versions or the use of different breeds. In particular, about 100 out of the 232 predicted miRNAs published by Capra et al. have been added to the latest miRBase release.

Regarding the genomic location of bovine sperm miRNAs, their distribution appears to be similar to the known distribution of cattle miRNAs published in miRBase and in good agreement with results in other species [72, 73], showing that miRNAs are frequently located within introns of protein-coding genes.

About 20% of sperm miRNAs have been located within genomic clusters (within 10 kb of another miRNA), in good agreement with estimates based on miRBase for cattle (22%) and humans (20%). We also explored whether genomically clustered miRNA are co-expressed to highlight physically linked miRNA, such as those produced from a same pri-miRNA transcript. Our analysis, based on statistical correlations, suggests that some, but not all (i.e., 20%), genomically clustered miRNAs may be co-expressed as a single polycistronic pri-miRNA transcript. Among the 43 clusters of co-expressed miRNA identified in this study, 15 were already described in humans [45]. Of note, miRNA belonging to the 9 remaining human clusters were not expressed in sperm. Our study thus expands the list of known polycistronic pri-miRNA clusters, adding in particular 5 large clusters ranging from 4 to 16 miRNAs. Interestingly, 8 clusters comprise predicted miRNAs alone, and two clusters comprise both known and predicted miRNA, strengthening their likelihood to be genuine miRNAs. Surprisingly, many strong positive and negative correlations were also observed between non-clustered miRNA pairs. Such correlations may correspond to regulatory networks, including for instance miRNAs co-regulated by shared regulatory features (positive correlation), as well as miRNAs hosted by transcripts targeted by other miRNAs (negative correlation). Regarding positive correlations, our analysis highlighted 77 correlation networks that mostly gather miRNAs differentially expressed between breeds, suggesting that they reflect mainly inter-breed genetic differences. Regarding negative correlation networks and assuming that intragenic miRNAs are transcribed in parallel with their host transcripts, we also identified putative miRNA–miRNA regulatory networks, encompassing 41 “master” miRNAs negatively correlated with other miRNAs hosted within their predicted targets. In support of this hypothesis, co-expression of miRNAs and their host genes has already been shown by expression studies [74, 75], as well as eQTL studies that show a substantial fraction of eQTLs shared between intragenic miRNAs and their host genes [76]. This mechanism is, however, far from general; many intronic miRNAs possess their own promoter and are transcribed independently of their hosting genes [77]. Additional data will be required to further explore in cattle whether miRNAs and their host genes are co-expressed, and in vitro transfection studies may be helpful in validating some of the putative miRNA–miRNA regulatory networks identified in this study.

A large number and an impressive diversity of isomiRs were also observed, with the number of isomiRs per miRNA varying greatly, ranging from a few up to one thousand. Some miRNA, such as miRNA-100, miRNA-146a, miRNA-151-3p, miRNA-143, miRNA-331-3p, miRNA-23b, miRNA-24, miRNA-222 and miRNA-199a-3p, have been proposed to be more prone to variations in human tissues [35]. Interestingly, these miRNAs also display a high number of isomiRs in our data (531 on average, compared to 97 for the whole miRNome). This suggests that common sequence features or conserved molecular mechanisms contribute to the isomiR profile across species. Surprisingly, published canonical sequences were found to be the most expressed in only half of the cases, and 14% of them were not detected at all in our data. Evolution of the criteria used to define canonical miRNAs throughout time [78], as well as tissue- and species-specific expression, may account for this situation.

### IsomiRs: an underexplored diversity

Focusing on 14,765 relevant isomiRs in terms of expression levels and representation across breeds, 3p variations appeared twice as frequently as 5p variations, both in terms of isomiR counts and expression. More than half of isomiRs are produced by a single 3p change (34%) or substitution within the miRNA sequence (21%), while single 5p changes account for only 8% and 38% result from a combination of several changes. These findings are consistent with previous reports, showing for instance that a vast majority of observed miRNA carried changes at their ends, in agreement with the hypothesis of cleavage variations by either Drosha or Dicer [43]. However, the high frequency of 3p changes, especially deletions, suggests that other mechanisms also contribute to the variation pattern. In particular, crystallographic studies have shown that 5p ends are tethered to Argonaute through multiple interactions and packed within the MID domain, whereas 3p ends may extend from the PAZ domain and may therefore be susceptible to 3′–5′ exonucleolytic activities [79]. We also described non-templated nucleotide additions, such as mono uridylation and adenylation, especially at 3p ends. This finding is consistent with other studies, showing that post-transcriptional modification of RNA and miRNA by 5p–3p nucleotidyl transferases, especially poly(A) polymerase and terminal uridylyl transferases, is a common mechanism, conserved in a variety of species [37, 80]. In cattle sperm, although mono-uridylation and adenylation affect a similar proportion of miRNA (32–35%), mono-uridylation seems to be the predominant mechanism, accounting for 34% of expression of isomiRs carrying 3p non-templated additions, compared to only 25% for mono adenylation.

We also identified substitutions as a major driver of miRNA variation, accounting for more than one-third of isomiRs. The vast majority of these polymorphic isomiRs are likely produced by miRNA editing, since only 6% of these variations co-localize with known SNPs. However, this percentage is probably underestimated due to limited availability of whole genome sequences from the Abondance, Charolais, Normand, and Belgian Blue breeds. Whole genome sequencing of bulls included in this study may help in the future to distinguish more precisely genetic polymorphism from variations introduced by editing. Several mechanisms have been described as major editing processes, including adenine and cytosine deamination (leading to A > G tor C > U transitions), nucleotide insertion in the mature miRNA by nucleotidyl transferases, and RNA-specific adenosine deaminase (ADAR) A > I deamination (detected as A > G transitions). Consistent with these data, our results showed an elevated frequency of C > U changes, the C substitution being 50% more frequent than expected based on nucleotide usage within miRNA sequences. A > G substitutions were also observed at lower frequency, suggesting that cytosine deamination is more frequent in bull sperm than adenine deamination. In our data, G > A and U > C transitions were observed at similar frequencies as A > G and C > U transitions. This suggests that in addition to classic A > I and C > U editing events, some other molecular mechanisms also significantly shape the sperm miRnome, making the editing paradigm much more complicated than initially thought. In line with our results, G > A and U > C changes have been reported in B lymphocytes [81], and G > A editing of WT1 transcripts has been associated with the APOBEC3A protein, a member of the APOBEC/ADAR protein family [82]. Interestingly, APOBEC3A has been shown to be expressed at high levels in mouse sperm heads [19] as well as human sperm, with upregulation between sperm achieving pregnancy and those that do not in intrauterine insemination [83]. Whether the relative frequency of classic vs non-classic editing in sperm may also be associated with APOBEC3A and impact bull fertility in cattle warrants future investigation. Furthermore, ADAR A > I editing has been previously described as the major driver of seed polymorphisms in the mouse brain, accounting for about 40% of changes within the seed [84]. This finding seems to be far from general, and in bull sperm G > A and C > U transitions are the most frequent changes within the seed. Polymorphisms within the seed and 5p variations together account for only 24% of isomiR expression. However, since polymorphisms as well as the seed region shift due to 5p variation may change the set of target mRNAs [39], these changes may be of functional importance, especially for isomiRs accounting for a large proportion of miRNA expression (e.g., 74 isomiRs polymorphic within the seed as well as 94 5p isomiRs having a relative expression above 50%). Overall, our findings on bull sperm support the notion that the diversity of expressed isomiRs should be considered instead of focusing on canonical miRNAs [78].

### Diversity of sncRNA profiles among breeds

Many sequences belonging to all sncRNA classes showed breed specific expression patterns ad were found to be differentially expressed between breeds. For instance, the Abondance and Monbeliarde breeds showed the highest number of differentially expressed sncRNAs, whatever the sncRNA class and the breed comparison, while only a few differential sequences could be identified between Holstein and Normande. The origin of these differences remains elusive and several hypothesis can be drawn. Indeed, breeds in this study differ in many aspects including genetic background (breed effect), local environment, nutrition and management, many of which may influence gene expression, making it difficult to decipher the breed and environment effect.

Consistent with a genetic origin of miRNA and piRNA differential expression between breeds, we observed changes in miRNA and piRNA expression according to sperm production parameters, which are under genetic control and are known to differ between breeds. In particular, Abondance bulls showed significant differences for motility related parameters and a large number of miRNA (833) and piRNA (5464) were found to be associated with these parameters, including miRNA [87] and piRNA (250) differentially expressed between Abondance and other breeds. Interestingly, a previous study already reported differentially expressed miRNA and piRNA between high and low motile spermatozoa [30]. About 25% of these miRNAs and 13% of piRNA located in these piRNA clusters were shown to be also associated with motility parameters in this study. This apparently low overlap between studies may result either from low statistical power or from physiological differences. Indeed Capra et al. compared two populations of high/normal and extremely low motile spermatozoa (i.e., VCL 100 vs 50 µm/s, ALH 3 vs 2.15 µm, Motility 48 vs 3.8%), whereas our study analyzed semen in a normal range of variation (i.e., VCL 280-150 µm/s, ALH 10-6 µm, Motility 90-25%).

Alternatively, environmental conditions may also influence sncRNA expression. In this respect, it is worth to note that Holstein and Normande bulls, which were housed in the same semen production center, also showed the lowest number of differentially expressed sncRNAs. Likewise, Charolais and Abondance bulls were housed in the same center and cluster close together based on miRNA expression despite differences in terms of semen functional parameters.

Disentangling genetic and environmental effect will require a large number of bulls form several breeds to be raised altogether in the same semen production center to evaluate the breed effect, and to duplicate this design in several semen production center to measure the environmental effect.

## Methods

### Semen collection

Commercial sperm straws were obtained for ten Holstein and Montbéliarde bulls, as well as five bulls for each of the following breeds: Normande, Charolais, Abondance and Belgian Blue. Thus a total of 40 representative bulls (normal sperm morphology and motility, good fertility) was selected for this study. Semen collection was performed by French and Belgian semen production centers (Evolution-XY, Umotest, Auriva-Elevage and AWE) following the routine protocol. Freshly ejaculated semen was diluted in egg yolk extender (Optidyl, IMV Technologies) before freezing using the DigitCool program (IMV Technologies).

### Total RNA isolation

Two straws per ejaculates (30–40 million sperm cells) were thawed and pooled for RNA extraction. Sperm quality was assessed after thawing using IVOS (Hamilton Thorne Inc) and flow cytometry (Easycyte 6HT, Guava Technologies Inc). Motility, kinetics, viability, mitochondrial potential activity and oxidation sensitivity were measured. A search for somatic cells was also performed using both visual examination under microscope (counting about 1000 sperm cells/sample) and flow cytometry (forward- and side- scatters), showing that contamination was below detectable levels. Remaining seminal plasma and extender were removed by washing the semen twice in 1 ml of Phosphate Buffered Saline solution (PBS), followed by centrifugation 5 min at 2400×*g*.

RNA extraction was performed according to an optimized guanidinium–Trizol total RNA extraction protocol, based on a guanidinium thiocyanate–phenol–chloroform extraction protocol [85]. Sperm pellets were homogenized in 100 µl of RLT buffer (Qiagen) supplemented with 1 µl beta-mercaptoethanol and incubated 15 min at room temperature. Then, 1 ml of Trizol (Invitrogen) was added and samples were incubated 5 min at room temperature. Complete lysis was ascertained by microscopic examination of 10 µl aliquots. After addition of 100 µl of chloroform and vigorous shaking for 15 s, samples were incubated 2 min at room temperature and centrifuged at 12,000×*g* during 15 min at 4 °C. The aqueous phase was transferred to a new collection tube and mixed with 100 µl of chloroform by vigorous shaking. After incubation for 2 min at room temperature and centrifugation at 12,000×*g* during 15 min at 4 °C, the aqueous phase was recovered and nucleic acids were precipitated overnight at − 20 °C using 1 volume of isopropanol supplemented with 25 µl glycogen (Ambion AM9510, 5 mg/ml). After centrifugation at 12,000 g during 15 min at 4 °C, pellets were washed with 75% ethanol and dried under vacuum. Dried pellets were then re-suspended in 12 µl of RNase free water and incubated 1 h at 4 °C before quality control assessment.

### RNA quality controls and sequencing

RNA concentration was assayed using the Qubit^®^ Fluorometer (Molecular Probes by Life Technologies) using the Qubit^®^ RNA HS Assay Kits (Q32852 Molecular Probes), according to the manufacturer’s recommendations. During protocol development, quality and concentration were also evaluated by electrophoretic assays on an Agilent Bioanalyzer 2100, using standard procedures.

In addition, RT-qPCR was used to quantify the expression of miRNA-125b-5p, which has been shown to be highly expressed in sperm [41]. Total RNAs (5 ng) were reverse transcribed in 10-μl reactions using the miRCURY LNA™ Universal RT microRNA PCR Starter kit (Exiqon). RNA spike-in control (Usp6) was also added during the RT-step. Triplicates were assayed in 10-μl qPCR reactions following the miRCURY LNA kit protocol, using a StepOnePlus Real time PCR System (Applied biosystems). Amplification curves were analyzed using the StepOne software v2.3 both for determination of Ct values and for melting curve analysis.

Library preparation and sequencing was done by the Exiqon service provider. Briefly, miRNA libraries were constructed using the NEBNext^®^ Multiplex Small RNA Library Prep Set for Illumina^®^ kit, including a size selection and quality control by Bioanalyzer. Deep sequencing was performed on Illumina HISeq2000, targeting 40 M single-reads (50 bp) per sample.

### Sequence analysis

Low-quality sequences were removed and adaptors were trimmed (Cutadapt). Sequences shorter than 17 nt were filtered and remaining sequences were then grouped by unique sequences to obtain total read counts for each. A workflow mostly based on miRDeep2 [86, 87] was used to identify and quantify known as well as predicted miRNAs. The unique sequences were mapped to the reference bull genome (Btau8, UMD3.1.1). Precursors and miRNAs were identified using the miRDeep2 core module, miRDeep2.pl. Potential miRNA datasets were created by adding known miRNA in bull (miRBase v22, March 2018) to miRNA associated with predicted precursors with a miRDeep2 score ≥ 0. The same operation was performed to create a data set of potential precursors. The quantifier.pl miRDeep2 module was then used to map unique reads, the set of potential miRNAs and all known miRNAs (miRBase v22) on the set of potential precursors enabling the annotation of miRNAs. The quantification results produced by the quantifier.pl module were then filtered with a custom perl script “parse_miRDeep2_outputs.pl” (https://github.com/SmartBioInf/PAQmiR) to eliminate any redundancy between known and predicted miRNAs.

**Fig. 16:**
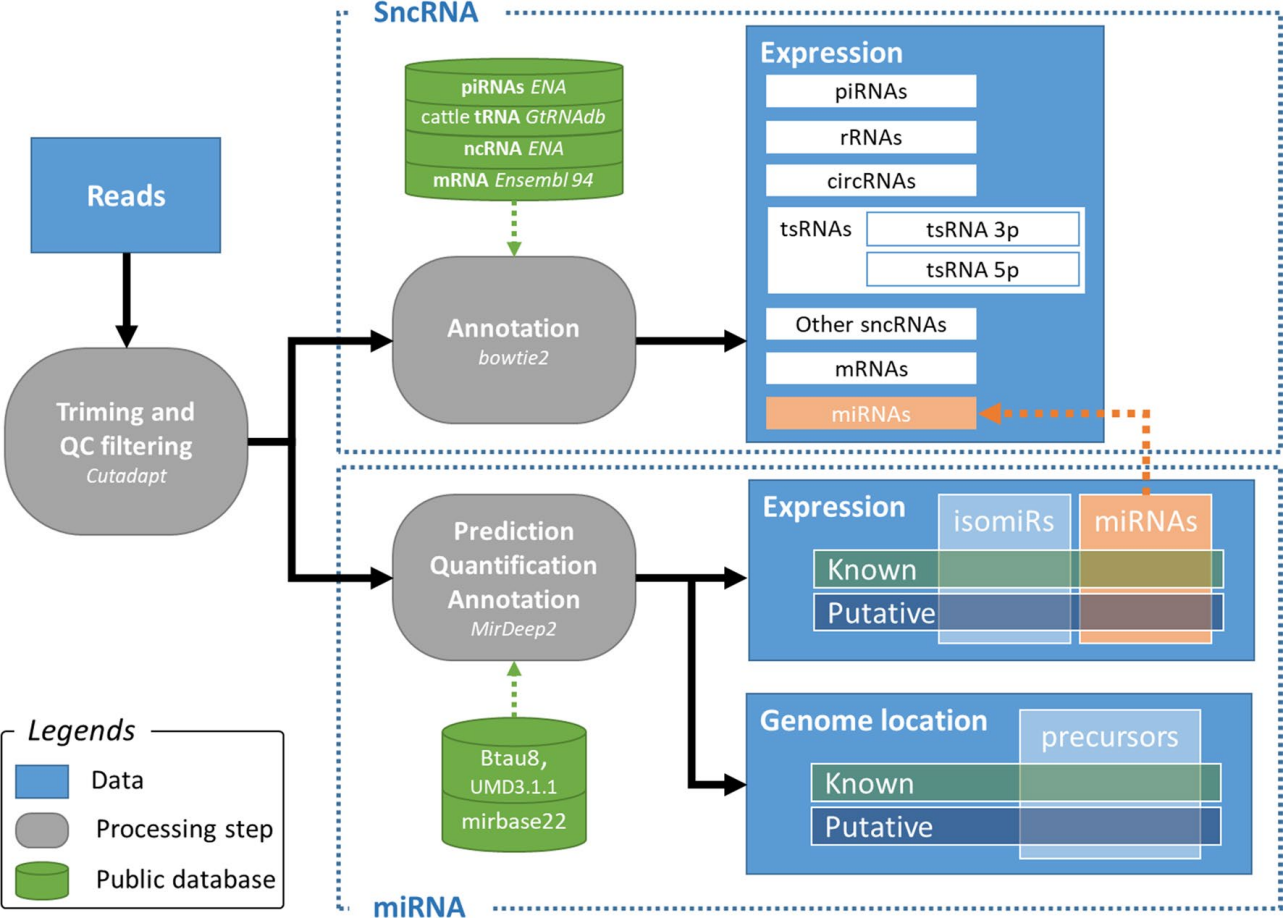
NGS data analysis workflow at a glance

Two separate expression files were generated from these results, providing either miRNA-level read counts (corresponding to similar sequences mapped to the same location), or isomiR-level read counts (corresponding to unique sequences). In parallel with this specific study of miRNAs, all unique sequences have been categorized by mapping to several databases and published data: cattle mRNA (Ensembl release 94, http://www.ensembl.org), RFAM release 14 (http://rfam.wustl.edu), cattle ncRNA from ENA (https://www.ebi.ac.uk/ena), cattle tRNA from GtRNAdb (http://www.gtrnadb.ucsc.edu), human, mouse and cattle piRNAs from ENA, as well as [30] and [88]. Figure 16 summarizes this analysis workflow.

An in-house pipeline [89] was used to annotate miRNA precursors relative to gene features (Ensembl release 94) and repeats (UCSC RepeatMasker file). The following criteria were applied for the delineation of promoters, transcription start sites (TSS) and transcription termination sites (TTS): promoter, − 2000 to + 100 bp relative to the TSS; TSS, − 100 to + 100 bp relative to the TSS; TTS: − 100 to + 100 bp relative to the TTS. The miRNAs located inside TTS, TSS, promotor, exon, intron, 3 or 5p UTR have been located in “genic” regions. On the other end, all miRNAs situated only on his own gene or in another place in the genome have been considered in “intergenic” regions.

Targetscan 7.2 (release March 2018) and Targetscan custom 5.2 were used to predict targets for known and predicted miRNAs, respectively. The gene ontology and KEGG pathways were explored using WebGestalt [90] (http://www.webgestalt.org/).

Correspondence between piRNA clusters published by Capra et al. and our piRNA sequences was ensured by mapping piRNA on cluster sequences using blast (options -task “blastn-short”), keeping only perfect matches along the whole piRNA sequence.

### RT-qPCR validation of breed differential expression

Two miRNA (bta-chr4_54509 and bta-mir-148b) and two isomiRs (bta-isomir-26a-1_1 and bta-isomir-26a-1_2) differentially expressed between Abondance and Normande breeds were chosen, as well as one miRNA differentially expressed between Charolais and Normande (bta-chr16_21053). LNA primers were purchased from Qiagen (sequences and references given in Additional file 3: Table S15). Additional bulls, which were not included in the RNA-Seq study, were chosen to perform the biological validation. Sperm RNA was produced for five bulls from each breed following the protocol described above. Total RNAs (5 ng) were reverse transcribed in 10-μl reactions using the miRCURY LNA™ Universal RT microRNA PCR Starter kit (Exiqon). Triplicates were assayed in 10-μl qPCR reactions following the miRCURY LNA kit protocol, using a StepOnePlus Real time PCR System (Applied biosystems). Amplification curves were analyzed using the StepOne software v2.3 to compute Ct values and analyze the melting curve. Relative expression was computed with the qbase^+^ software (Biogazelle).

### Statistical analysis

Expression similarity between miRNA pairs was assessed with the Pearson correlation matrix, using the *rcorr* and *p*.*adjust* functions from the Hmisc R package. Only miRNAs with mean normalized expression greater than 10 in at least one breed were included in the correlation study. The Circos plot was drawn using the RCircos R package. Cytoscape 3.7.1 [91] was used to build correlation networks, using the Prefuse Force Directed layout. Between-class correspondence analysis was performed using the ade4 R package (with the *dudi.bca* function). DESeq 2 [92] was used to normalize read counts and test for differential sncRNA expression between breeds, based on negative binomial generalized linear models. Statistical significance was evaluated based on Benjamini–Hochberg adjusted *p*-values [93], with a significance threshold of 5%. Multiple comparison tests between semen functional parameters were performed using the wilcox_test function from the R rstatix package and boxplots were drawn using the ggboxplot function from the ggpubr package. Correlations between semen functional parameters and miRNA and piRNA expression was performed using the *rcorr* and *p*.*adjust* functions from the Hmisc R package. Correlations above |0.7| (*p* value ≤ 0.1) were kept for miRNA, while correlations above |0.8| (*p* value ≤ 0.001) were kept for piRNA.

### Data availability

The (trimmed) raw data have been stored at ENA, European Nucleotide Archive, under the primary accession number: PRJEB33940.

## Conclusion

Our study provides a comprehensive overview of cattle sperm sncRNAs, and our findings will pave the way for future work on the functional significance of sncRNAs in terms of semen quality and fertility. In particular, the role of rRNAs, tRFs and isomiRs at fertilization and during zygote development warrants future study. In particular, their individual expression levels, global expression profile, or frequency of particular editing events may prove to be relevant biomarkers for sperm quality or bull management, and thus of great interest for artificial insemination companies.

## Supplementary information

**Additional file 1: Fig S1.** RNA quality control. Bioanalyzer profile and RT-qPCR with miRNA-125b-5p primers were performed to validate RNA size, quality and concentrations, starting from 5 ng of total RNA, as estimated by Qubit^®^ RNA HS Assay Kit. (**a**) Typical electrophoretic profiles were obtained, with the expected dome around 20 nucleotides and no evidence of 18S and 28S rRNAs. (**b**) Consistent amplification results were obtained (Ct in the range 20–21), indicating that the Qubit –estimated concentration was reliable and that no inhibitors remain in the RNA preparation. Single peak melting curves were also obtained, indicating that a single, specific product has been produced.

**Additional file 2: Fig S2.** NGS data quality controls. **(a)** Phred Quality score distribution over all sequences for the 10 first samples, showing Q score above 30 for more than 97% of reads. **(b)** Read length distribution for the 10 first samples, showing two main peaks at 18–26 nt (microRNA or siRNA) and 28–32 nt (piRNA or tsRNAs.

**Additional file 3: Table S1.** The Sperm miRnome. Catalogue of miRNAs of bovine frozen sperm. **Table S2.** Sperm rRNAs. Catalogue of rRNAs of bovine frozen sperm. **Table S3.** Sperm tRNAs. Catalogue of tRNAs of bovine frozen sperm. **Table S4.** Sperm piRNAs. Catalogue of piRNAs of bovine frozen sperm. **Table S5.** Other sperm small RNAs. Catalogue of other small non coding RNA (without rRNA, tRNA and piRNAs) **Table S6.** Sperm mRNAs. List of reads corresponding to mRNA fragments of ejaculated bovine sperm. **Table S7.** Table of miRNAs expression in bovine frozen sperm. **Table S8.** miRNA genomic clusters. List of genomic clusters identified in bovine sperm. **Table S9.** Expression correlations between miRs. **Table S10.** Expression correlations between miRs located in genomic clusters. **Table S11.** Expression of sperm isomiRs across 6 cattle breeds. **Table S12.** Distribution of isomiRs expression levels and contribution to miRNA expression (%). **Table S13.** Details (expression and changes) about isomiRs accounting for more than 1% of each miRNA expression level. **Table S14.** DeSeq 2 results. List of sncRNAs differentially expressed between breeds. **Table S15.** RTqPCR validation: list of LNA primers, normalized expression and statistics. **Table S16.** Sperm functional parameters across breeds. **Table S17.** Comparison of DEmiR across breeds and DEmir published in Capra et al. **Table S18.** Correlation of sperm parameters with miRNAs and piRNAs expression across breeds. **Table S19.** Comparison of miRNA content and expression with Capra et al.

**Additional file 4: Fig S3.** Reads annotated as mRNA fragments. About 24,992 reads have been identified as mRNA fragments. **(a)** The vast majority of these reads were 50 nt in length. **(b)** IGV profiles of two genes (AKAP1 and PRM1) covered by well distributed unique reads having high count levels.

**Additional file 5: Fig S4.** Reads annotated as rRNA. Distribution of reads across 18S **(a)** and 28S **(b)** rRNAs show a particular pattern made of several peaks and read-poor sub regions, suggesting that rRNAs are fragmented by selective RNA cleavage.

**Additional file 6: Fig S5.** Biogenesis of tRFs and classification of fragments. The cloverleaf structure of a tRNA typically contains a D-Loop, an Anticodon-Loop, a variable loop, a T-loop, and an amino acid acceptor stem. **(a)** Several endonucleases can cleave tRNAs at specific sites, generating tRFs of different categories: cleavages of an RNase at the D-loop or T-loop of a tRNA can generate a 5′ or 3′ tRFs, respectively. Dicer (DCR) endonucleases have been reported to cleave tRNA at D-Loop, T-Loop and the amino acid acceptor stem. RNase Z cleavage has been shown to produce tRF3s n a Dicer-independent manner. Moreover, Angiogenin (ANG), a member of the RNase A superfamily, was shown to cleave the Anticodon-Loop to produce tRNA halves upon stress stimuli. ANG is also able to cleave the T-loop of tRNAs. Other unknown RNases might also participate in tRFs generation. **(b)** Multiple tsRNAs alignments along the tRNA4174-LeuAAG sequence (D-Loop in red, T-Loop in yellow, Variable-Loop in green and amino acid acceptor stem in violet), illustrating the five tRFs categories: tRF5 and tRF3 (~ 15–32 nt fragments), 5′-tRHs and 3′-tRHs (30–35 nt) and i-tRFs. Reads that didn’t fall into these categories were classified as other, possibly including full-length tRNAs (limited to the first 50th nucleotides due to the sequencing protocol). Post-transcriptional modifications and/or genetic polymorphisms may also affect tRFs (nucleotide depicted in red). Criteria used to define the categories are depicted below the multiple alignment. Full line indicates the mandatory region that should be covered by the tRF, while dot line indicate the range that can be covered by the Trf.

**Additional file 7: Fig S6.** Frequency of tRFs isotypes. The percentage of read counts was computed for each tRF and each associated anticodon, according to the tRF length. Only the most expressed tRFs are reported in the histogram. The left axis (0–40%) refers to 5p-tRHs, which are the most expressed tsRNAs. The right axis (0–10%) refers to the other tsRNAs.

**Additional file 8: Fig S7.** Distribution of cleavage sites along the anticodon-Loop, for both 5p-tRHs and 3p-tRHs, showing a bias towards 5p of the anticodon and a high frequency of cleavage at the 4^th^ and 7^th^ nucleotides.

**Additional file 9: Fig S8.** Distribution of Pearson correlation for all and clustered miRNAs. Correlation coefficients were computed for the top 1580 miRNAs having a mean expression level above 10 in at least one breed (all miRNA) and clustered miRNAs. Correlations of a miRNA with itself were omitted. Stronger correlations were observed between genomically clustered miRNA compared to non-clustered miRNAs.

**Additional file 10: Fig S9.** Five putative functional regulation networks. A search was performed to identify specific patterns of correlation (above |0.7|) indicative of putative functional regulation (miR-x - > miR-y - > miR-z implies negative correlation between miR-x and miR-y as well as miR-y and miR-z, while a positive correlation is expected between miR-x and miR-z).

**Additional file 11: Fig S10.** Classification and examples of isomiRs for bta-chr29_51574. Multiple alignments along the Genome and the Pre-miRNA sequence are shown, illustrating the diversity of changes occurring in isomiRs and clarifying the nomenclature used in the text.

**Additional file 12: Fig S11.** Frequency of substitution and nucleotide usage in miRNA sequences. The frequency of each nucleotide was computed based on all canonical miRNA sequences and compared to the frequency of substitution in isomiRs. Ratio above or under 1 are suggestive of non-random substitutions. Substitutions involving C and U appeared to be 50% and 10% more frequent than expected by chance, while G substitutions appeared 20% less frequent than expected.

## Data Availability

The (trimmed) raw data have been stored at ENA, European Nucleotide Archive, under the primary accession number: PRJEB33940.
