# Supplementary figures and images for: A comprehensive overview of bull sperm-borne small non-coding RNAs and their diversity across breeds
Source: Epigenetics Chromatin. 2020 Mar 30;13:19. doi: 10.1186/s13072-020-00340-0 (PMC7106649; doi:10.1186/s13072-020-00340-0)

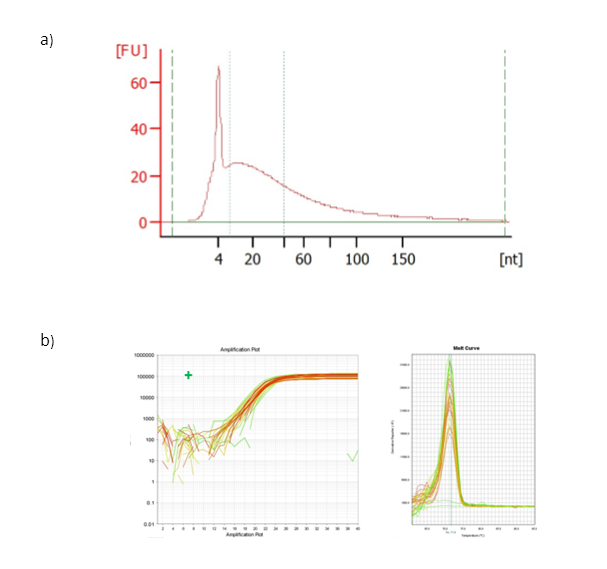

Supplement: Supplementary file 1 — Additional file 1: Fig S1. RNA quality control. Bioanalyzer profile and RT-qPCR with miRNA-125b-5p primers were performed to validate RNA size, quality and concentrations, starting from 5 ng of total RNA, as estimated by Qubit® RNA HS Assay Kit. (a) Typical electrophoretic profiles were obtained, with the expected dome around 20 nucleotides and no evidence of 18S and 28S rRNAs. (b) Consistent amplification results were obtained (Ct in the range 20–21), indicating that the Qubit –estimated concentration was reliable and that no inhibitors remain in the RNA preparation. Single peak melting curves were also obtained, indicating that a single, specific product has been produced. [file 13072_2020_340_MOESM1_ESM.tif]

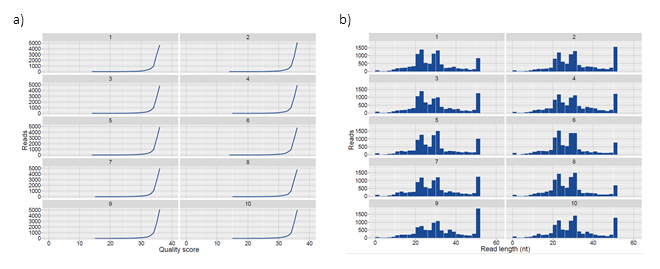

Supplement: Supplementary file 2 — Additional file 2: Fig S2. NGS data quality controls. (a) Phred Quality score distribution over all sequences for the 10 first samples, showing Q score above 30 for more than 97% of reads. (b) Read length distribution for the 10 first samples, showing two main peaks at 18–26 nt (microRNA or siRNA) and 28–32 nt (piRNA or tsRNAs. [file 13072_2020_340_MOESM2_ESM.tif]

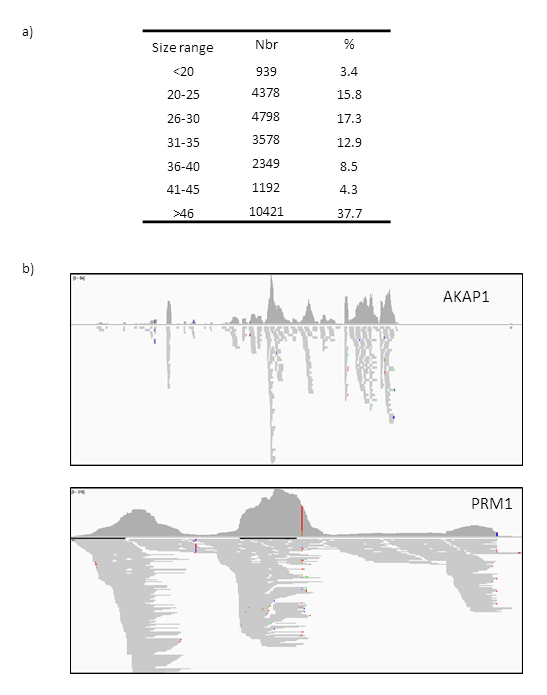

Supplement: Supplementary file 4 — Additional file 4: Fig S3. Reads annotated as mRNA fragments. About 24,992 reads have been identified as mRNA fragments. (a) The vast majority of these reads were 50 nt in length. (b) IGV profiles of two genes (AKAP1 and PRM1) covered by well distributed unique reads having high count levels. [file 13072_2020_340_MOESM4_ESM.tif]

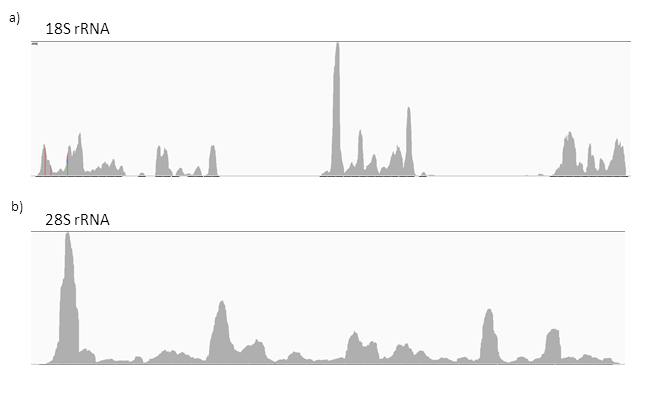

Supplement: Supplementary file 5 — Additional file 5: Fig S4. Reads annotated as rRNA. Distribution of reads across 18S (a) and 28S (b) rRNAs show a particular pattern made of several peaks and read-poor sub regions, suggesting that rRNAs are fragmented by selective RNA cleavage. [file 13072_2020_340_MOESM5_ESM.tif]

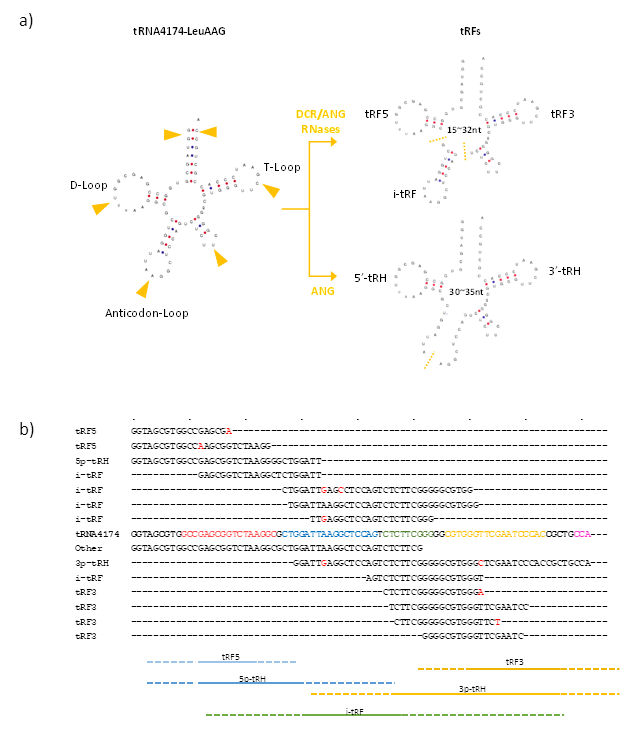

Supplement: Supplementary file 6 — Additional file 6: Fig S5. Biogenesis of tRFs and classification of fragments. The cloverleaf structure of a tRNA typically contains a D-Loop, an Anticodon-Loop, a variable loop, a T-loop, and an amino acid acceptor stem. (a) Several endonucleases can cleave tRNAs at specific sites, generating tRFs of different categories: cleavages of an RNase at the D-loop or T-loop of a tRNA can generate a 5′ or 3′ tRFs, respectively. Dicer (DCR) endonucleases have been reported to cleave tRNA at D-Loop, T-Loop and the amino acid acceptor stem. RNase Z cleavage has been shown to produce tRF3s n a Dicer-independent manner. Moreover, Angiogenin (ANG), a member of the RNase A superfamily, was shown to cleave the Anticodon-Loop to produce tRNA halves upon stress stimuli. ANG is also able to cleave the T-loop of tRNAs. Other unknown RNases might also participate in tRFs generation. (b) Multiple tsRNAs alignments along the tRNA4174-LeuAAG sequence (D-Loop in red, T-Loop in yellow, Variable-Loop in green and amino acid acceptor stem in violet), illustrating the five tRFs categories: tRF5 and tRF3 (~ 15–32 nt fragments), 5′-tRHs and 3′-tRHs (30–35 nt) and i-tRFs. Reads that didn’t fall into these categories were classified as other, possibly including full-length tRNAs (limited to the first 50th nucleotides due to the sequencing protocol). Post-transcriptional modifications and/or genetic polymorphisms may also affect tRFs (nucleotide depicted in red). Criteria used to define the categories are depicted below the multiple alignment. Full line indicates the mandatory region that should be covered by the tRF, while dot line indicate the range that can be covered by the Trf. [file 13072_2020_340_MOESM6_ESM.tif]

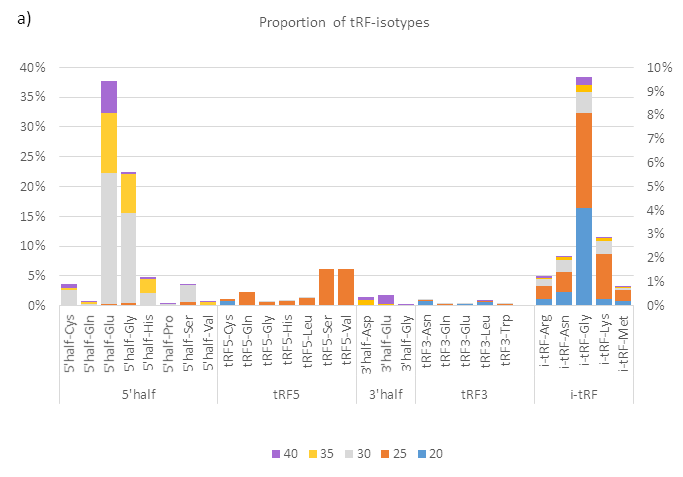

Supplement: Supplementary file 7 — Additional file 7: Fig S6. Frequency of tRFs isotypes. The percentage of read counts was computed for each tRF and each associated anticodon, according to the tRF length. Only the most expressed tRFs are reported in the histogram. The left axis (0–40%) refers to 5p-tRHs, which are the most expressed tsRNAs. The right axis (0–10%) refers to the other tsRNAs. [file 13072_2020_340_MOESM7_ESM.tif]

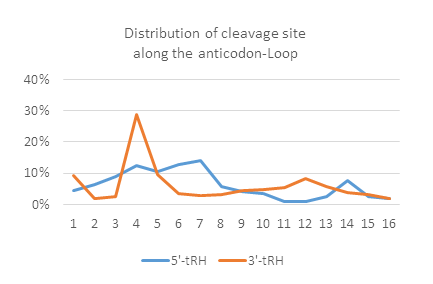

Supplement: Supplementary file 8 — Additional file 8: Fig S7. Distribution of cleavage sites along the anticodon-Loop, for both 5p-tRHs and 3p-tRHs, showing a bias towards 5p of the anticodon and a high frequency of cleavage at the 4th and 7th nucleotides. [file 13072_2020_340_MOESM8_ESM.tif]

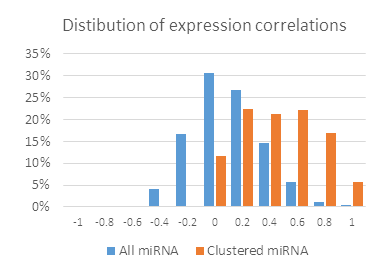

Supplement: Supplementary file 9 — Additional file 9: Fig S8. Distribution of Pearson correlation for all and clustered miRNAs. Correlation coefficients were computed for the top 1580 miRNAs having a mean expression level above 10 in at least one breed (all miRNA) and clustered miRNAs. Correlations of a miRNA with itself were omitted. Stronger correlations were observed between genomically clustered miRNA compared to non-clustered miRNAs. [file 13072_2020_340_MOESM9_ESM.tif]

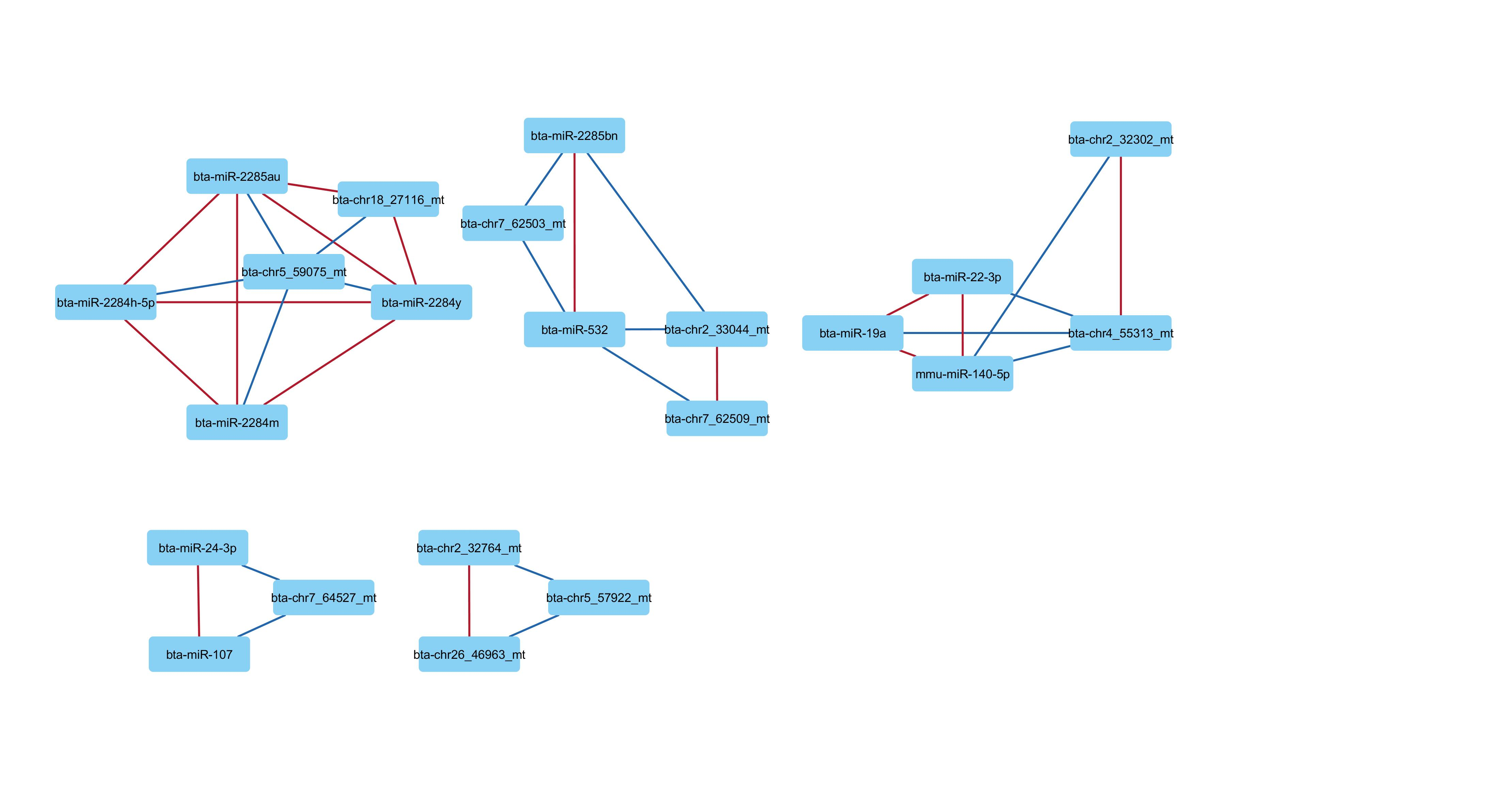

Supplement: Supplementary file 10 — Additional file 10: Fig S9. Five putative functional regulation networks. A search was performed to identify specific patterns of correlation (above |0.7|) indicative of putative functional regulation (miR-x - > miR-y - > miR-z implies negative correlation between miR-x and miR-y as well as miR-y and miR-z, while a positive correlation is expected between miR-x and miR-z). [file 13072_2020_340_MOESM10_ESM.tif]

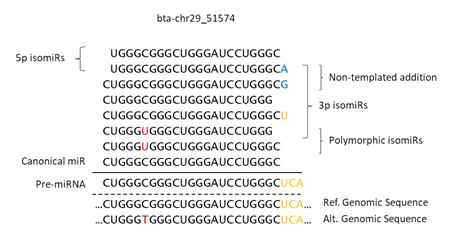

Supplement: Supplementary file 11 — Additional file 11: Fig S10. Classification and examples of isomiRs for bta-chr29_51574. Multiple alignments along the Genome and the Pre-miRNA sequence are shown, illustrating the diversity of changes occurring in isomiRs and clarifying the nomenclature used in the text. [file 13072_2020_340_MOESM11_ESM.tif]

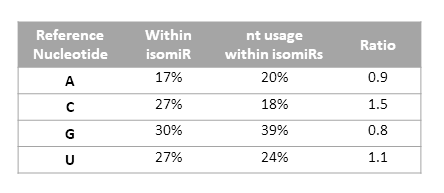

Supplement: Supplementary file 12 — Additional file 12: Fig S11. Frequency of substitution and nucleotide usage in miRNA sequences. The frequency of each nucleotide was computed based on all canonical miRNA sequences and compared to the frequency of substitution in isomiRs. Ratio above or under 1 are suggestive of non-random substitutions. Substitutions involving C and U appeared to be 50% and 10% more frequent than expected by chance, while G substitutions appeared 20% less frequent than expected. [file 13072_2020_340_MOESM12_ESM.tif]
